# The Importance of Mitochondrial Processes in the Maturation and Acquisition of Competences of Oocytes and Embryo Culture

**DOI:** 10.3390/ijms26094098

**Published:** 2025-04-25

**Authors:** Elżbieta Gałęska, Alicja Kowalczyk, Marcjanna Wrzecińska, Mercedes Camiña García, Ewa Czerniawska-Piątkowska, Szymon Gwoździewicz, Wojciech Witkiewicz, Zbigniew Dobrzański

**Affiliations:** 1 Department of Environment Hygiene and Animal Welfare, Wrocław University of Environmental and Life Sciences, 51-630 Wrocław, Poland; elzbieta.galeska@upwr.edu.pl (E.G.); marcjanna.wrzecinska@zut.edu.pl (M.W.); zbigniew.dobrzanski@upwr.edu.pl (Z.D.); 2 Department of Physiology, University of Santiago de Compostela, 15705 Santiago de Compostela, Spain; merchi.camina@usc.es; 3 Department of Ruminant Science, West Pomeranian University of Technology in Szczecin, 70-310 Szczecin, Poland; ewa.czerniawska-piatkowska@zut.edu.pl; 4 Institute of Animal Breeding, Wrocław University of Environmental and Life Sciences, 51-630 Wrocław, Poland; 5 Research and Development Center, Voivodeship Specialist Hospital in Wrocław, 51-124 Wrocław, Poland; witkiewicz@wssk.wroc.pl

**Keywords:** mitochondria, maturation, oocytes, embryo culture, reproduction

## Abstract

Mitochondria, as multifunctional and partially independent structures, play a crucial role in determining essential life processes. Recently, their significance in reproductive biology has gained increasing attention. This review aims to comprehensively analyse the role of mitochondrial processes in oocyte maturation and embryo culture. A comprehensive literature review was conducted to highlight the importance of mitochondrial activity in the early stages of life formation. Proper mitochondrial function provides energy, maintains genomic stability, and ensures optimal conditions for fertilisation and embryo progression. Understanding these processes is essential to optimise culture conditions and identify new mitochondrial biomarkers that improve reproductive success and improve assisted reproductive technologies (ARTs). Enhancing mitochondrial function in female reproductive cells is the key to improving oocyte and embryo quality, which can lead to better in vitro fertilisation and embryo transfer. Furthermore, advances in diagnostic techniques, such as mitochondrial genome sequencing, offer a more precise understanding of the relationship between mitochondrial health and oocyte quality. However, fully understanding mitochondrial functions is only part of the challenge. Expanding knowledge of the interactions between mitochondria and other cellular structures is crucial for future advancements in reproductive medicine. Understanding these complex relationships will provide deeper insight into improving reproductive outcomes and embryo development.

## 1. Introduction

Mitochondria play a key role in cellular metabolism, particularly in the production of energy in the form of adenosine triphosphate (ATP) through cell respiration by oxidative phosphorylation (OXPHOS) and the electron transport chain (ETC) [1,2]. These structures are highly dynamic and enclosed within a bilayer lipid membrane [3]. The outer mitochondrial membrane (OMM) is made of a lipid bilayer with embedded proteins that participate in mitophagy, while the second layer, the inner mitochondrial membrane (IMM), is specialised for cellular respiration [3,4]. Both membranes are separated by the mitochondrial intermembrane space (IMS) [5]. The OMM participates in the transport of substances into or out of the mitochondrion [3]. The IMM, in turn, forms cristae that increase the surface area of the organelle and serve as a matrix for the OXPHOS pathway [1]. The ETC complexes are integrated into the inner mitochondrial membrane [6], and the IMM also transports enzymes such as superoxide dismutase (SOD) and glutathione peroxidase (GPx). The intermembrane space plays a role in apoptosis and in the import of proteins necessary for the maintenance of mitochondrial DNA (mtDNA) [7]. In the mitochondrial matrix, processes such as carbohydrate and lipid catabolism, the tricarboxylic acid cycle (TCA; also known as the Krebs cycle) and oxidative phosphorylation (OXPHOS) occur, which then leads to energy generation [7]. Mitochondria, as cellular centres of energy in the cell, are 0.5–10 μm in size and contain their own mitochondrial genome, which is associated with the energy demand [8,9].

**Figure 1:**
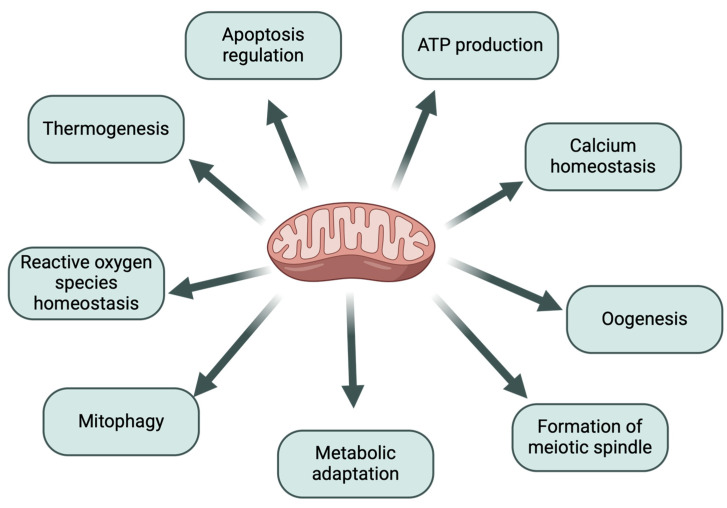
Mitochondria functions (created with Biorender.com (accessed on 17 March 2024).

In addition to their role in cellular metabolism, mitochondria are also closely linked to maintaining homeostasis, which is achieved through a dynamic balance of apoptosis, mitochondrial autophagy, fission, and fusion [10]. During apoptosis, damaged cells are eliminated. Mitochondrial autophagy is essential for maintaining mitochondrial homeostasis by degrading dysfunctional mitochondria. Furthermore, mitochondrial fission and fusion, working in coordination with mitochondrial autophagy, are key processes that regulate mitochondrial homeostasis [10], and fusion is necessary to maintain cellular metabolism processes [11]. Moreover, they are involved in calcium homeostasis [12]. The most important functions of mitochondria are presented in Figure 1.

Mitochondria are also involved in the storage of mRNA, which occurs with the help of MARDO in oocytes from various species, including mice, cattle, pigs, and humans [13]. According to Cheng et al. [13], MARDO is a mitochondrial-associated ribonucleoprotein domain that forms around mitochondria and is regulated by an increase in mitochondrial membrane potential during the development of oocytes. As the oocyte develops, both mitochondria and MARDO are stored together in the ooplasm. Mitochondria serve as a platform for the assembly of MARDO, which, in turn, may influence the distribution of mitochondria. The formation of MARDO depends on the increase in mitochondrial membrane potential during oocyte growth. During the growth phase of the oocytes, the mitochondria maintain relatively low activity, which results in reduced ROS production and less DNA damage, promoting the stability of the genetic material in oocytes. In oocytes of mice, cattle, pigs, and humans, MARDO acts as a structural linker that holds mitochondria together, and its disruption leads to their dispersion. MARDO shares some similarities with the Balbiani body: both MARDO and the Balbiani body cluster mitochondria and may support active mitochondria. The Balbiani body is believed to play a role in selecting active and healthy mitochondria. Interestingly, the formation of MARDO also depends on the increase in mitochondrial membrane potential during oocyte growth. However, MARDO and the Balbiani body differ in several ways. First, they appear at different stages of oocyte development—the Balbiani body forms early in oocyte development, while MARDO appears in later stages. Second, they are composed of different materials—the Balbiani body is maintained by an amyloid-like matrix, while MARDO, being a linked structure, has hydrogel properties and exchanges with the cytosol. Additionally, the Balbiani body contains the Golgi apparatus, while MARDO does not interact with it. Nevertheless, both MARDO and other RNA-containing, membrane-less compartments share some common components [13].

Mitochondria are often referred to as the ‘powerhouses’ of the cell, playing a crucial role in reproduction, including fertilisation and embryo development, due to the high energy demand during these processes [14,15]. ATP is crucial for the process of oocyte maturation, fertilisation, and subsequent embryo development [16,17]. In the study by Ge et al. [18], it was shown that mitochondrial functions influence reproductive success. They also participate in the formation of the meiotic spindle, breaking down the germinal vesicles [19,20,21]. Furthermore, the oocyte contains an exceptionally dense population of mitochondria, in which mitochondrial DNA plays a crucial role. A decrease in mtDNA, for instance, has been linked to ovarian ageing [14,22]. Human mtDNA covers ~16,569 base pairs [23]. It is estimated that a mitochondrion contains 2–10 copies of mtDNA [24], of which growth occurs during oogenesis, and a mature metaphase II oocyte contains ~500,000 copies of the mitochondrial genome [25]. Therefore, disturbances in mitochondrial function can lead to reproductive disorders and low oocyte quality [26,27].

The aim of this review was to determine the importance of mitochondrial processes in the maturation and acquisition of competences of oocytes and in the culture of mammalian embryos.

### 1.1. The Importance of Mitochondria in the Process of Oogenesis

Oogenesis is a key process for reproductive success [28]. At birth, ovaries contain a reserve of oocytes surrounded by granulosa cells, which form primordial follicles. These remain dormant until activation, when the folliculogenesis process begins and leads to an approximately 100-fold increase in oocyte volume [29]. Oocyte growth and maturation are associated with the accumulation of mRNA [30], proteins, and cellular organelles—including mitochondria [30,31]. During the maturation process of mammalian oocytes, they progress from the germinal vesicle (GV) stage to metaphase II [32]. After the disintegration of the GV, the meiotic spindle forms, which facilitates the segregation of the chromosomes. Mitochondria provide the necessary energy for processes such as spindle formation and polar body extrusion (PBE), which are highly energy-consuming [32,33,34,35].

Additionally, morphological changes in mitochondria are observed as they take the form of oval structures with well-defined cristae [29]. During oogenesis, oocytes increase their volume by nearly 300-fold, which is closely associated with mitochondrial accumulation [15]. Mitochondrial dysfunction at this stage results in insufficient energy supply, reducing oocyte quality and leading to improper chromosome segregation, which ultimately causes embryo aneuploidies [29,32,36]. Consequently, mitochondria are considered markers of oocyte quality [29]. Mitochondria are generated during oogenesis, but their replication is halted until the embryo reaches the blastocyst stage [37].

Studies conducted in mice have demonstrated that during oocyte maturation, mitochondria undergo dynamic changes [15]. In the germinal vesicle stage, mitochondria were characterised by an even distribution within the ooplasm. However, as the oocytes progressed to the MI and MII stages of the gametes, the organelles aggregated, with this effect intensifying as the cells matured [15]. The study confirmed that the number of mtDNA copies increases with oocyte maturation. In addition, cells with improperly distributed mitochondria and their reduced dynamics are characterised by poorer quality, lower ATP levels, and consequently reduced developmental potential [9].

An important factor that affects fertility, particularly oocyte quality, is the presence of diseases such as diabetes [38]. Studies conducted by Wang et al. [39] demonstrated that oocytes from diabetic mice exhibited reduced ATP levels and spindle defects, resulting in abnormalities in chromosome segregation compared to oocytes obtained from healthy females [39]. This indicates mitochondrial dysfunction as a consequence of the disease, contributing to the generation of reactive oxygen species (ROS). These, in turn, lead to oxidative stress, causing damage to nucleic acids and triggering cell apoptosis [38].

Furthermore, other studies have shown that Mfn1 knockout in mouse oocytes results in female infertility and disrupted folliculogenesis [40]. The authors also observed structural changes in the mitochondria of Mfn1-null oocytes compared to the non-knockout group [40]. Mitofusin 1 and 2 (Mfn1, Mfn2) are proteins that regulate mitochondrial fusion in mammalian cells, ensuring proper organelle function [9]. Both Mfn1 and Mfn2 are present in the outer mitochondrial membrane [41] and play a role in calcium homeostasis, OXPHOS subunit activity, and energy supply during various stages of cell development [42,43]. Mitofusins are also critical in mitophagy—the process of removing damaged mitochondria [9].

### 1.2. The Relationship Between Mitochondrial Function and Oocyte Quality

Mature oocytes do not produce mitochondria, and the generation of these organelles resumes during embryo development to the blastocyst stage [15]. However, during dormancy, they maintain metabolic activity, making the proper functioning of mitochondria crucial for their viability [44]. During this phase, mitochondrial complex I is inactive, but other OXPHOS complexes remain functional, which helps to prevent excessive ROS production and the resulting oxidative stress [45]. For ovulation, oocytes require ATP, and the necessary metabolic substrates are supplied by cumulus cells and follicular fluid [46]. Any alteration in the structure or quantity of mitochondria directly affects the quality of oocytes, developmental competence, and subsequent implantation of mammalian embryos [15]. In turn, a reduction in oocyte quality leads to a decrease in female fertility.

Moreover, due to their critical role in calcium homeostasis, mitochondria also participate in calcium oscillations, which are essential for activation during fertilisation [47]. Maintaining proper mitochondrial function and intracellular Ca^2+^ concentration influences the development potential of oocytes [48]. Studies conducted in mouse oocytes demonstrated that calcium fluctuations occur at two critical moments: before polar body extrusion and after its extrusion, coinciding with readiness for cell activation and requiring significant energy input [47]. Increased levels of mitochondrial calcium are essential for the activation of enzymes in the Krebs cycle and the electron transport chain, which subsequently improves ATP production [49].

The research conducted by Cheng et al. [48] investigated the impact of intracellular calcium levels on the developmental competence of mouse oocytes following cryopreservation. The oocytes were classified into experimental groups, where one group was treated with 2-aminoethoxydiphenyl borate (2-APB) to block the release of calcium ions, and another group was treated with thapsigargin (TG), an inhibitor of calcium pump proteins in the endoplasmic reticulum (ER). A control group consisting of untreated oocytes was also included. Each group consisted of fresh oocytes from the same mouse randomly assigned to the control group, of which approximately half were cryopreserved and warmed. The findings revealed that TG-treated oocytes showed higher calcium levels, which interfered with cell survival and blastocyst formation. On the contrary, oocytes exposed to 2-APB maintained stable calcium levels. The researchers concluded that cryopreservation has a negative effect on calcium concentrations and causes damage to organelles, which affects the developmental competence of oocytes [48].

### 1.3. The Relationship Between Mitochondrial Function and Embryo Quality

The quality of embryos is largely determined by the quality of oocyte mitochondria, as the mitochondria carried by sperm after fertilisation are degraded within the ooplasm [46,50]. The mitochondrial genome in preimplantation embryos undergoes dynamic changes, with the highest levels of OXPHOS activity and mitochondrial membrane potential reached at the blastocyst stage [51]. Various processes, from fertilisation to cell division and blastocyst hatching, can generate oxidative stress, which is typically mitigated by mitochondrial enzymes [46]. Energy, essential for embryonic development, is generated in mitochondria from pyruvate, lactate, glucose, and amino acids [52,53,54,55]. Pyruvate is a key nutrient for the development of embryos from the zygote stage to the 2-cell stage [53,56]. It also serves as the primary energy source for preimplantation embryos, where it is converted into acetyl-CoA (acetyl coenzyme A) through oxidative phosphorylation [57]. From the morula stage, glucose is integrated into the glycolysis pathway [46], which is crucial for providing energy to germ cells and developing embryos [56]. The conversion of pyruvate to acetyl-CoA is mediated by pyruvate dehydrogenase (PDH), whose activity is regulated by pyruvate dehydrogenase kinase (PDK) and pyruvate dehydrogenase phosphatase. PDK activity inhibits PDH, leading to the accumulation of pyruvate, while high levels of PDH promote the conversion of pyruvate to acetyl-CoA, resulting in ATP production in the TCA cycle and OXPHOS [56,57].

According to research conducted by Zhang et al. [49], reducing the concentration of pyruvate in the culture medium inhibited the development of mouse embryos at the 2-cell stage in vitro and also decreased the presence of mitochondria. Furthermore, the authors showed that culturing embryos with 0.2 times the control pyruvate concentration (0.2 mM pyruvate) reduced ROS levels compared to the control group. In early developmental stages, embryos exhibit low metabolic activity to avoid ROS-induced oxidative stress, but excessively low levels of reactive oxygen species can disrupt redox balance and may lead to developmental arrest [52].

Similar studies were carried out in human embryos [58], which were subjected to in vitro maturation (IVM) in Earle’s medium with 0.47 mmol pyruvate as the control and experimental groups with 0.23 mmol and no pyruvate. In each group, the glucose concentration was maintained at 1 mmol. The results showed that the removal of pyruvate from the medium resulted in a reduction in blastocyst formation, with only 4 out of 25 embryos in this group reaching the blastocyst stage. Additionally, a decrease in metabolic activity of embryos was observed [58].

Other studies [57] examined the relationship between pyruvate metabolism and epigenetic regulation in bovine embryo culture. Dichloroacetate (DCA), a pyruvate analogue, was used to inhibit pyruvate dehydrogenase kinase and block the phosphorylation of pyruvate dehydrogenase, thus inhibiting the conversion of pyruvate to acetyl-CoA. Iodoacetate (IA), which inhibits glyceraldehyde-3-phosphate dehydrogenase (GAPDH), was also used to block glycolysis. After 8 h of incubation, both groups (DCA and IA) exhibited a reduced mitochondrial membrane potential and lower levels of acetyl-CoA, as well as decreased mitochondrial membrane potential in blastocysts. The IA group showed impaired glycolysis and decreased acetyl-CoA levels, leading to disruptions in the TCA cycle and reduced mitochondrial membrane potential. DCA, on the other hand, increased the conversion of pyruvate to acetyl-CoA, which then entered the pentose phosphate pathway instead of glycolysis [43]. Furthermore, the decrease in acetyl-CoA availability has been shown to alter the pattern of histone acetylation, which may lead to impaired molecular control. Reduced acetyl-CoA levels may result from mitochondrial overload and increased reactive oxygen species, which hinder embryo development [57].

Pawlak et al. [59] analysed the impact of metabolic disturbances during the in vitro maturation of bovine oocytes on the quality of the resulting blastocysts. The study was carried out in cumulus–oocyte complexes (COCs) subjected to IVM in control groups (without inhibitors) and experimental groups treated with iodoacetate (IO) as a glycolysis inhibitor, DHEA (pentose phosphate pathway inhibitor), and ETOMOXIR (fatty acid metabolism inhibitor). After IVM, the oocytes were fertilised in vitro (IVF), and the blastocysts were evaluated for the lipid composition of the blastocysts on day 8. The results showed a significant inhibition of lipid metabolism in the experimental groups compared to the control group, as well as a reduction in the number of blastomeres. These findings suggest that disturbances in glucose and fatty acid metabolism negatively affect the quality characteristics of developing blastocysts [59]. Furthermore, the research confirmed that environmental factors such as stress and maternal physiology influence mitochondrial function in preimplantation embryos [51].

## 2. Metabolic Processes in Mitochondria

In oocyte energy metabolism, cumulus cells play a crucial role since oocytes themselves are incapable of glucose uptake. Cumulus cells metabolise glucose through processes such as anaerobic glycolysis and the pentose phosphate pathway (PPP) [56,60,61]. Granulosa cells convert glucose into lactate via pyruvate. However, the activation of pyruvate dehydrogenase kinase can inhibit the OXPHOS pathway, leading to a reduction in ATP production and limiting cell proliferation. During PPP, the activity of glucose-6-phosphate dehydrogenase activity increases, resulting in greater synthesis of NADPH. Elevated NADPH levels subsequently promote the production of ribose and deoxyribose, which are essential for nucleic acid synthesis [61].

Because of its structure, glucose cannot freely cross the phospholipid bilayer of biological membranes. Specialised glucose transporters (GLUTs), which are transmembrane proteins, are required to facilitate its transport into the cell [56]. In the female reproductive system, the cooperation between cumulus cells, oocytes, and gap junctions leads to the formation of cumulus–oocyte complexes (COCs) [56].

Glycolysis occurs in the cytoplasm and generates two ATP molecules per glucose molecule [61]. The products of glycolysis, such as pyruvate [62], are transported into oocytes via paracrine signalling and gap junctions [60]. Monocarboxylates, such as lactate and pyruvate, are transported into oocytes through monocarboxylate transporters (MCTs), while pyruvate is transferred to the mitochondrial matrix by the mitochondrial pyruvate carrier (MPC) [61].

In mitochondria, pyruvate undergoes oxidation by pyruvate dehydrogenase (PDH), resulting in the production of acetyl coenzyme A. Acetyl-CoA combines with oxaloacetate (OAA) to form citrate, the first intermediate in the TCA cycle [63]. Through this process, oocytes generate energy in the form of ATP through the Krebs cycle and oxidative phosphorylation [60].

Additionally, oocytes acquire free fatty acids from follicular fluid and cumulus cells, but they can also synthesise these compounds independently. Free fatty acids are transported into mitochondria, where they participate in β-oxidation and can also be stored as neutral triglycerides (TAGs). Subsequently, these fatty acids are integrated into the TCA cycle and OXPHOS [60]. Furthermore, β-oxidation of fatty acids positively influences the process of oocyte maturation [61].

### 2.1. Oxygen Metabolism

**Figure 2:**
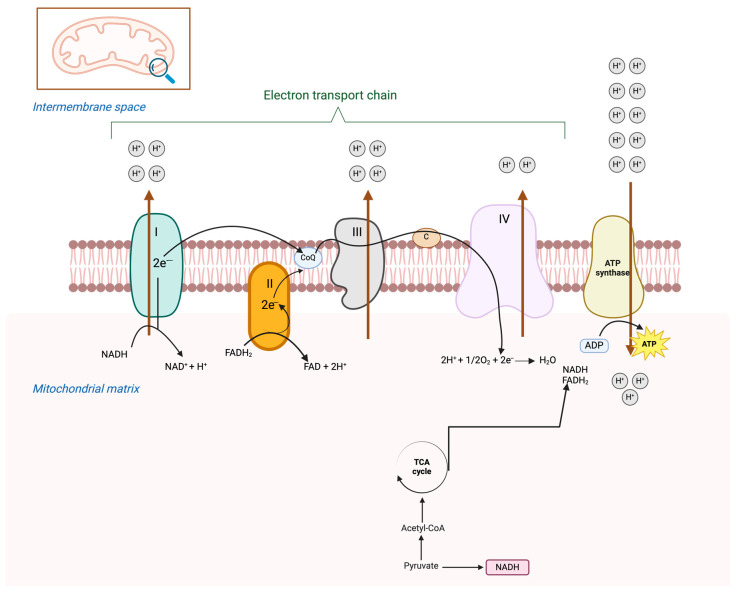
Electron transport chain. Legend: CoQ—CoQ10; C—cytochrome C (created with Biorender.com (accessed on 28 January 2025)).

Mitochondria play a crucial role in cellular metabolism, primarily through the electron transport chain [64]. The catabolic process of glucose in cells begins with glycolysis, which produces pyruvate. This molecule is then incorporated into mitochondria as a substrate for oxidative phosphorylation, which occurs during the tricarboxylic acid cycle [65]. Electrons generated during this cycle are transported through NADH and FADH_2_ to ETC complexes located on the inner mitochondrial membrane (Figure 2) [66]. During this process, electrons are transferred along the membrane, enabling the pumping of protons into the intermembrane space through a mechanism known as oxidative phosphorylation. The resulting proton gradient drives ATP synthase, which converts ADP into ATP [67,68]. This metabolic pathway (OXPHOS) ultimately produces 36 molecules of ATP [61].

The respiratory complexes in the ETC function as proton pumps, utilising the energy derived from electrons to increase the electrochemical potential across the inner mitochondrial membrane [69]. However, it is important to note that electron transport is not entirely efficient, as electrons may ‘leak’ in complexes I and III. This leakage leads to the formation of reactive oxygen species, such as the superoxide anion radical [70,71]. This radical is converted into hydrogen peroxide (H_2_O_2_) by superoxide dismutases: SOD1 in the mitochondrial intermembrane space and SOD2 in the mitochondrial matrix [72,73]. Subsequently, H_2_O_2_ is further neutralised by enzymes such as glutathione peroxidase (GPx) and glutathione reductase (GR), with NADPH acting as a reducing equivalent [73]. Excess ROS production can disrupt the redox balance within the cell, leading to mitochondrial dysfunction and oxidative damage to membrane lipids, proteins, and highly sensitive mtDNA, which is not protected by histones [32,71].

### 2.2. Lipid Metabolism

Mitochondria can utilise various substrates as energy sources. In the case of glucose depletion, lipids are incorporated into metabolism [74]. In mammalian oocytes, energy is stored in the form of lipid droplets that contain triglycerides [75,76]. Lipids, including fatty acids (FAs), glycerides, phosphoglycerides, steroids, sphingolipids, and lipoproteins, serve as an alternative energy source and a building material for cellular structures [77]. Lipid synthesis occurs mainly in the endoplasmic reticulum, although mitochondria also play a significant role in their metabolism [78].

Mitochondria are involved in fatty acid β-oxidation, a process aimed at energy production. β-oxidation is regulated by cytoplasmic and mitochondrial enzymes. The key enzymes involved in this process are fatty acid synthase (FASN), ATP citrate lyase, acyl-CoA synthetase, and carnitine acetyltransferase [77]. The oxidation process of β-oxidation occurs in mitochondria or peroxisomes and leads to the production of acetyl-CoA, which is later incorporated into the tricarboxylic acid cycle (Krebs cycle) [76,79]. Additionally, this process generates NADH and FADH_2_, which play a crucial role in the electron transport chain [76].

Mitochondria have the ability to synthesise fatty acids through the action of cytoplasmic and mitochondrial enzymes, with palmitic acid being the final product. Subsequently, fatty acids can be utilised for the construction of phospholipid membranes, including mitochondrial membranes, ensuring their proper potential. The loss of mitochondrial capacity to metabolise and synthesise lipids can disrupt the assembly of the electron transport chain, directly affecting energy production [80]. A reduction in oocytes’ ability to oxidise fatty acids, which increases with age, can lead to disturbances in ATP synthesis. Impaired β-oxidation efficiency results in elevated cellular stress, which consequently reduces the quality of gametes and embryos during the peri-implantation period [60].

### 2.3. Ca^2+^ Metabolism

Mitochondria play a vital role in the regulation of calcium signalling and the maintenance of intracellular Ca^2+^ homeostasis [81]. They function as reservoirs for calcium ions and are involved in their release [82]. Under normal physiological conditions, Ca^2+^ acts as a signal to stimulate ATP production by activating mitochondrial dehydrogenase enzymes [83]. This calcium signalling further impacts various metabolic pathways, while calcium ions released from the endoplasmic reticulum into mitochondria can regulate mitochondrial enzyme activity, directly shaping the organelle’s metabolism. Any disruption in calcium levels caused by diseases or stress can affect redox balance within the cell [71].

Calcium ions are essential for processes such as cell division [84], autophagy [85], differentiation [86], and ageing [87]. Excessive mitochondrial calcium uptake can lead to the generation of reactive oxygen species, triggering inflammation and apoptosis [88].

The concentration of calcium ions in the extracellular matrix is several times lower than in the intracellular space due to distinct regulatory mechanisms [83]. The highest calcium concentrations are found in the endoplasmic reticulum, which serves as the main intracellular calcium store [89]. In cellular terms, mitochondria and ER are calcium stores [90]. In the endoplasmic reticulum, calcium levels range between 500 and 1000 μM, which is significantly higher than in the cytosol [91]. The ER membrane contains numerous calcium channels, including ryanodine receptors (RyRs) and inositol 1,4,5-triphosphate receptors (IP3Rs). On the contrary, calcium ions are transported out of the ER by Ca^2+^-ATPase pumps [89].

In mitochondria, calcium transport occurs through the voltage-dependent anion channel (VDAC) in the outer mitochondrial membrane, which facilitates calcium permeability, and through the mitochondrial calcium uniporter (MCU) in the inner mitochondrial membrane (IMM) [92]. The MCU complex is regulated by proteins such as EMRE, MICU1, MICU2, MICU3, MCUb, and MCUR1, which collectively control calcium ion flow. MICU1-3 proteins, in particular, bind calcium ions and modulate MCU activity [93]. Calcium transfer between the ER and mitochondria is mediated by IP3R receptors [94]. This process results in transient calcium concentration spikes in mitochondria, with the signal propagating through the MCU. Subsequently, calcium ions reach the outer mitochondrial membrane, the intermembrane space, and ultimately contribute to the formation of the negative IMM potential dependent on Ca^2+^ ions [94].

Under physiological conditions, fluctuations in calcium levels are triggered by muscle contractions or hormonal stimulation [91]. In mitochondria, calcium levels are critical for energy production, as they influence the activity of mitochondrial matrix dehydrogenases [91,94]. Research conducted by Katona et al. [94] demonstrated that excessive calcium uptake by mitochondria can lead to overload, resulting in increased ROS production, inhibition of ATP synthesis, and cytochrome c release. This calcium overload in neuronal mitochondria contributes to cell death, as observed in a mouse model of Alzheimer’s disease [95].

Calcium homeostasis dysregulation is also a risk factor for neurodegenerative diseases, ischemic stroke, and chronic heart conditions, including heart failure [96,97,98]. During episodes of oxygen and glucose deprivation, ATP production is disrupted, leading to membrane depolarisation and activation of voltage-gated calcium channels. This, in turn, causes excitotoxicity, mitochondrial dysfunction, increased ROS levels, and ultimately apoptosis [99].

## 3. Mitochondrial Changes

Mitochondria are essential for energy production, supporting metabolism, and maintaining cellular homeostasis [11]. However, various factors can cause mitochondrial dysfunction, including genetic mutations, ageing, and physical inactivity [100]. Although inherited mitochondrial mutations are rare (1 in 5000 individuals), they can lead to severe neurological disorders, such as myopathies [101]. Mutations in the mitochondrial genome, for example, can affect cardiolipin (CL), a phospholipid essential for mitochondrial membranes and their processes, which can potentially lead to cardiomyopathy [100].

Bacterial infections, such as those caused by *Listeria monocytogenes* [102] and *Helicobacter pylori* [103] can induce mitochondrial fragmentation, while viruses like HIV promote mitophagy, disrupting mitochondrial balance and damaging neurons [100]. Additionally, physical inactivity promotes metabolic diseases such as diabetes and contributes to mitochondrial dysfunction, including a reduction in mitochondrial number within muscle cells [104].

Neurodegenerative diseases (NDs), such as Alzheimer’s and Parkinson’s disease, are associated with the accumulation of misfolded proteins in neuronal cells, altering the nervous system [105]. Patients with NDs exhibit elevated levels of reactive oxygen species, which generate oxidative stress. This, in turn, damages mitochondria and nucleic acids, and promotes β-amyloid aggregation, a hallmark of Alzheimer’s disease [106,107]. Similarly, oxidative stress and mitochondrial dysfunction also play a critical role in oocyte ageing and embryo development, potentially compromising fertility and embryonic viability.

Hypoxia (deprivation of oxygen) is another key factor that affects mitochondrial function, particularly the respiratory chain. Studies in human endothelial cells [108] derived from the umbilical vein showed that 6-day exposure to hypoxia (1% O₂) increased the expression of hypoxia-inducible factor 1α (HIF1α), a marker of oxygen deficiency [108]. Additionally, the activity of antioxidant enzymes such as superoxide dismutase and glutathione reductase (GR) decreased by approximately 25% and 60%, respectively. Hypoxia caused an increase in ROS levels, with mitochondrial ROS nearly doubling compared to the control group. Furthermore, hypoxia led to a reduced level of coenzyme Q10 (Q10), a crucial mitochondrial antioxidant, resulting in a decrease in the efficiency of oxidative phosphorylation and ATP production [108].

AMP-activated protein kinase (AMPK) plays a vital role in maintaining mitochondrial function [109]. It is a serine/threonine protein kinase that is a regulator of cell energy homeostasis, including glucose and lipid metabolism [55]. Studies conducted in mouse oocytes (5–6 weeks old) analysed the influence of AMPK on pre-ovulatory ageing of oocytes. Old oocytes were characterised by lower expression of AMPK, contributing to increased ROS levels and mitochondrial dysfunction [110].

The expression of mitochondrial genes is also regulated by mTOR (mammalian target of rapamycin), a serine-threonine kinase involved in autophagy, cell growth, and metabolism [111]. mTOR controls the expression of cytochrome c, a key protein in mitochondrial processes [112].

Mitochondrial sirtuins (SIRT3-5), which are NAD+-dependent deacetylases, also play a critical role in mitochondrial function. SIRT3 interacts with ATP synthase and contributes to the potential of the mitochondrial membrane. Reduced levels of SIRT3 result in decreased activity of mitochondrial enzymes, increased ROS production, and oxidative stress. Meanwhile, SIRT5 supports NADPH homeostasis and antioxidant activity [112].

### 3.1. Ageing Processes in Mitochondria

Ageing is a process influenced by both internal and external factors, such as diet, body weight, and environmental conditions [113]. It is well known that female reproductive abilities decline with age, which is associated with a decrease in the quality of the ovarian reserve and a decrease in the number of primordial follicles [37,60]. Older oocytes exhibit chromosomal aberrations, spindle apparatus damage, zona pellucida, and organelle damage [114,115]. Cellular ageing is a complex process driven by various factors, such as oxidative stress, telomere shortening, and DNA damage that contribute to mutations. Ageing cells also show changes in their morphology [7,116]. In older individuals, iron homeostasis is often disrupted, predisposing them to cardiovascular and neurodegenerative diseases, which are linked to oxidative damage [116]. This occurs due to iron overload, which generates hydroxyl radicals, increasing the peroxidation of polyunsaturated fatty acids in cells and leading to apoptosis [116]. Moreover, ageing results in reduced mitochondrial function [115].

Ageing is also associated with an increase in reactive oxygen species, which negatively impacts mitochondrial function, leading to mutations in mtDNA, oxidative damage to proteins and organelles, and shortening of the telomere. The accumulation of these negative effects leads to mitochondrial apoptosis, which is crucial for oocyte function. Mitochondrial dysfunction results in energy deficits necessary for cellular processes [115,117]. In older patients, a decrease in the number of mitochondria and the degradation of their cristae is often observed. These mitochondria are generally elongated and enlarged and can adopt irregular shapes [118]. The morphology also changes with age [118]. A known mediator of the ageing process is the protein p53, which affects mitochondrial dynamics and promotes their elongation [7]. Ovarian ageing has been shown to affect mitochondrial morphology, resulting in the vacuolisation of organelles and impairing their function [37,119]. Additionally, the calcium storage capacity of the endoplasmic reticulum in ageing oocytes deteriorates, leading to a disruption of calcium homeostasis, which, in turn, reduces calcium signalling during fertilisation and oocyte maturation, finally affecting gamete quality [115].

Ageing is associated with a reduction in the efficiency of the mitochondrial electron transport chain, leading to a decrease in ATP levels, which ultimately lowers oocyte quality [60]. It is also associated with an increase in ROS content, which is a by-product of metabolic processes in mitochondria that produce energy [37]. When the REDOX balance is disturbed, ROS have a detrimental impact on mitochondria, leading to oxidative damage, including damage to mtDNA. This can result in follicular atresia, significantly reduce the fertilisation potential of oocytes, or cause female infertility [26,44]. Oxidative stress leads to an imbalance between free radicals and antioxidants, resulting from the intense production of ROS in mitochondria. This causes oxidative damage to the mitochondrial genome and reduces fertilisation rates [114].

Studies conducted in mouse oocytes analysed ROS and ATP levels in mitochondria [115]. For this purpose, the oocytes obtained from older animals at the age of 44–48 weeks were to correspond to oocytes aged 45–60 years in the perimenopausal period of women and oocytes from younger mice (6–8 weeks). Older oocytes were characterised by a decrease in the extrusion of the first polar body, abnormalities of the meiotic spindle, higher levels of ROS, and lower levels of ATP in relation to the younger group of younger oocytes. Furthermore, during the study, the level of expression of the critical calcium-binding protein (CABL1) was also analysed by analysing the expression of the calbindin 1 gene (Calb1). This protein is important in the context of regulating cellular calcium levels, which translates into the regulation of metabolism and apoptosis. The researchers analysed the level of this protein in mouse oocytes for the first time, indicating its protective role during oocyte ageing [115]. It was proven that the expression of the tested protein is crucial during oocyte maturation and maintains a constant level, which decreases as cells age. During this examination, the researchers detected lower levels of CALB1 protein expression in old MII stage oocytes compared to the young group, which may contribute to gamete ageing. Furthermore, an attempt was made to silence the CABL1 encoder gene using siRNA targeting *Calb1*. For this purpose, the designed siRNA was injected into the ooplasm, and PBS was injected into the control group. The results showed that siRNA treatment caused a decrease in CABL1 protein, and thus, a 60% reduction in the production of the polar body by oocytes was achieved, and it also showed abnormalities in the meiotic spindle. The authors proved that the reduced expression of the tested protein in oocytes was associated with a decrease in calcium concentration in the ER and mitochondria. A decrease in mitochondrial membrane potential was also detected, which indicated impaired organelle functions [115]. Moreover, the overexpression of CABL1 effectively corrects age-related oocyte defects. It improves spindle assembly and chromosome alignment while restoring calcium homeostasis in the ER and mitochondria. Additionally, it reduces oxidative stress and increases ATP levels. The enhanced mitochondrial function further contributes to improved fertilisation rates and embryo development. These findings suggest that CABL2 plays a key role in counteracting oocyte ageing. In their study, the authors showed that CABL1 participates in the regulation of oocyte ageing [115].

Sirtuins, a family of protein deacetylases dependent on nicotinamide adenine dinucleotide (NAD+) and ADP-ribosyltransferases, play a crucial role in regulating the ageing process and protecting oocytes from oxidative stress [117]. These proteins act as regulators of various cellular processes, including apoptosis, DNA repair, and the maintenance of energy and redox homeostasis [117]. As the reproductive system ages, the expression levels of SIRT1-3 in oocytes decrease, while the expression of SIRT4 and SIRT6 increases [60]. As individuals age, NAD+ levels decrease [120], leading to a reduction in the cofactor available for sirtuins, which consequently results in a decrease in their activity [121]. Studies have shown that SIRT1 improves mitochondrial function, repairs DNA damage, and participates in oocyte maturation, but its activation is diminished by oxidative stress [122]. Research in mouse oocytes has demonstrated that SIRT1-3 protects oocytes from ageing after ovulation by mitigating the effects of ROS and abnormal mitochondrial distribution [123]. In these studies, oocytes were exposed to nicotinamide (NAM) as an inhibitor of SIRT1, SIRT2, and SIRT3 at concentrations of 0, 1, 5, and 10 mM. After 6 h of incubation, ROS levels were assessed. At higher concentrations of NAM (5 and 10 mM), an increase in ROS was observed, leading to the induction of ageing. Furthermore, NAM-treated oocytes showed altered spindle morphology, with gradual loss of microtubules and changes in mitochondrial distribution compared to the control group, where mitochondria exhibited a polarised distribution pattern. These results suggest that SIRT1-3 are involved in protecting oocytes from ageing [123].

Other studies by Xing et al. [122] on mouse oocytes analysed the function of SIRT1 in the ageing process. The oocytes were obtained from mice and divided into fresh cells (without incubation) and aged cells (12 h of incubation in M16 medium). The RNA was then extracted, and the SIRT1 coding sequence was sequenced for subsequent injection into the MII oocytes. Control-aged oocytes were injected with PBS. The authors found a decrease in SIRT1 expression in aged oocytes. Furthermore, SIRT1-injected oocytes showed an increase in protein expression compared to the control group. A decrease in ROS levels was also observed with increased SIRT1 expression, which may indicate the modulation of superoxide dismutase and the mitigation of the effects of ageing [122].

The purpose of the study conducted by Pasquariello et al. [124] was to determine the possible correlation between mitochondria function and number and the ATP content and functionality of the meiotic spindle in oocytes obtained from young (at 8 weeks) and older (52–56 weeks) mice. The biological material was divided into three groups: control (oocytes of young mice), study (oocytes of old mice), and those obtained from older mice matured in vitro with the addition of antioxidants. Old oocytes were characterised by a lower mitochondrial membrane potential than those obtained from younger animals. Furthermore, the antioxidant supplementation of older oocytes contributed to the achievement of a mitochondrial DNA number comparable to the number of mtDNA copies in young oocytes, while old oocytes without supplementation were characterised by a reduced number of copies. Researchers obtained similar results by analysing the ROS level in older oocytes; it was higher than in the control sample and the one tested with the addition of antioxidants. The results indicate a negative impact of female ageing on mitochondrial function [124]. Thus, ageing is directly related to a reduction in mitochondrial function [125].

### 3.2. Thermal Stress

Warm-blooded organisms are susceptible to heat stress under certain environmental conditions when the threshold of their thermoneutral zone is exceeded [126]. Studies have shown that heat stress reduces milk production in cows [127], alters poultry meat quality [128], decreases feed intake in animals [129], and negatively impacts reproductive parameters by lowering the developmental potential of oocytes and disrupting mitochondrial functions [130].

Heat stress (HS) induces oxidative stress, which damages mitochondria, reducing their functionality [131]. HS contributes to lipid peroxidation in mitochondrial membranes, leading to increased production of reactive oxygen species. The response to HS involves a cascade of gene activation, particularly those encoding heat shock proteins (HSPs), which stabilise ROS-damaged proteins [131]. HSPs act as molecular chaperones and are essential to maintain cellular processes [132]. Among these proteins, HSP70 and HSP90 play a significant role and are expressed in response to heat stress [133]. HSP70 is involved in stabilising the cytoskeleton, regulating the cell cycle, and preventing apoptosis [134], while HSP90 is crucial to maintaining cellular homeostasis [132]. The expression of HSPs protects cells against hyperthermia and aims to preserve their functions during stress [135].

Mitochondria are particularly sensitive to heat stress, which disrupts key processes such as cell respiration, energy production, and calcium homeostasis [126,131,136]. Heat stress also leads to changes in mitochondrial morphology, which often causes them to swell [137]. In oocytes exposed to heat stress, ROS accumulation occurs, reducing their developmental potential [130]. Mitochondrial dysfunction includes impaired ATP synthesis and the disruption of spindle assembly [138]. The imbalance in REDOX homeostasis caused by heat stress results in excessive ROS accumulation, increasing mitochondrial membrane permeability and leading to cytochrome C release, ultimately causing cell damage and apoptosis [137]. Damaged mitochondria are removed through mitophagy, a form of mitochondrial autophagy, which prevents excessive ROS accumulation and further damage [139]. Heat stress negatively affects reproductive processes by inhibiting follicular growth, reducing oocyte quality, suppressing steroidogenesis, and generating ROS, which adversely affects embryonic development [130,140].

### 3.3. Exposure to Toxic Substances

The relationship between environmental toxins and mitochondrial functions is being explored [141,142,143,144]. To analyse the impact of toxic substances on mitochondria, researchers examine the number of mtDNA copies [145]. Polycyclic aromatic hydrocarbons (PAHs) are lipophilic toxic substances that can accumulate in mitochondria and bind to mtDNA. In a study conducted by Jia et al. [146], to evaluate the impact of PAHs on mtDNA, blood samples were collected from 19 oil field workers. Significant changes in the methylation of the *COX* gene (cytochrome c oxidase) were observed at two loci—*MT-COX1* and *MT-COX2*—while no differences were detected at the *MT-COX3* locus. Increased methylation of the mitochondrial genome may lead to dysfunction of these organelles. The *COX* gene is involved in the electron transport chain and encodes complex IV. Mutations in this gene can lead to ETC dysfunction [146].

Moreover, PAHs contribute to the generation of reactive oxygen species and oxidative damage [147]. Wang et al. [148] investigated the effect of phenanthrene (PHE) on the quality of mouse oocytes. The gametes were cultured with PHE at concentrations of 200, 300, and 400 μM, as well as without PHE (control group). The study revealed that PHE disrupted oocyte maturation and polar body extrusion and caused spindle multipolarity with abnormal structure in treated oocytes. On the contrary, the spindles in the control group exhibited normal morphology and barrel-shaped shapes. Furthermore, chromosomes in PHE-treated oocytes were misaligned and improperly arranged. PHE also increased intracellular calcium levels, demonstrating mitochondrial dysfunction and ROS accumulation [148].

Microplastics are currently being widely studied for their health impacts due to their components, such as polyethylene and polystyrene [149]. Both microplastics and nanoplastics can absorb toxic metals, intensifying their negative effects [150]. Toxic metals can enter the body through food, water, or air [151]. These metals, such as lead, mercury, cadmium, arsenic, and cobalt, are persistent pollutants that do not degrade [152,153]. They cause cytotoxicity and damage mitochondria by disrupting the REDOX balance [154].

Wu et al. [150] analysed the effect of polystyrene nanoplastics (PS-NPs) and cadmium on rat ovaries. Rats were assigned to groups receiving daily doses of 2 mg/kg PS-NPs, 1.5 mg/kg Cd, a combination of 1.5 mg/kg Cd and 2 mg/kg PS-NPs, or a control group (no exposure) for four weeks. Subsequently, the rats were euthanised, and their ovaries were collected. Histological analysis revealed that the groups exposed to toxic substances exhibited a reduction in growth follicles and a higher proportion of atretic follicles compared to the control group. In each treated group, increased ROS levels were detected, with the highest levels observed in the cadmium and nanoplastic combination group, while the levels of SOD and CAT were significantly lower than in the control group. Additionally, loss of mitochondrial cristae and membrane rupture were observed in the cadmium and nanoplastic group, leading to mitochondrial dysfunction [150]. Exposure to toxic metals is associated with increased mitochondrial membrane permeability, respiratory chain complex damage, and elevated levels of reactive oxygen species [144].

**Figure 3:**
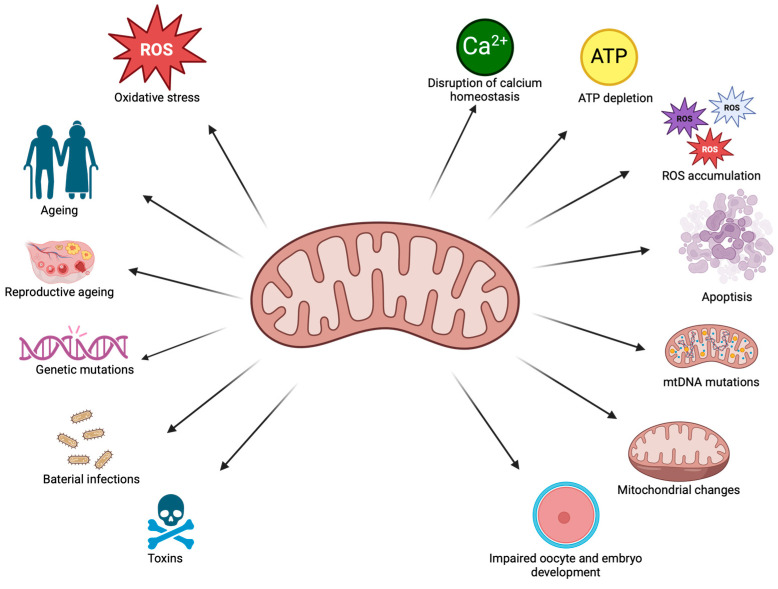
Mitochondrial changes (created with Biorender.com accessed on 29 March 2025).

A schematic representation of the factors influencing mitochondrial dysfunction and their impact on cellular processes is presented in Figure 3.

## 4. Methods of Regenerating Mitochondria

Mitochondria are subject to control in the form of biogenesis and mitophagy [112]. The way to restore mitochondria in cells is through their biogenesis (mitobiogenesis), which takes place simultaneously with mitophagy, that is, the elimination of mitochondria [155]. Mitobiogenesis occurs in the case of increased demand for energy and other cellular signals [155]. Methods of restoring mitochondrial function can also include antioxidant supplementation or mitochondrial replacement therapy (MRT) [156,157].

### 4.1. Mitochondrial Replacement Therapy (MRT)

The quality and proper functioning of mitochondria are crucial for oocyte maturation and embryonic development, and abnormalities result in disorders such as premature ovarian ageing (POA) or diminished ovarian reserve (DOR) [32]. To limit the inheritance of mitochondrial diseases and improve the quality of oocytes, mitochondrial replacement therapy is used, which includes mitochondrial transfer, which aims to increase the number of mitochondria in oocytes, the ooplasm exchange method, and transfer of the meiotic spindle, pronucleus, and polar body [50,158,159]. In the case of spindle transfer, the spindle–chromosome complex is transferred from the mother’s oocyte to the donor oocyte with properly functioning mitochondria. Transfer of the pronucleus involves simultaneous fertilisation of the donor and recipient oocytes and then the transfer of the pronucleus from the zygote to the healthy enucleated donor oocyte. In turn, polar body transfer involves transferring it instead of the spindle to the enucleated recipient oocyte with correct mitochondria [160]. Although the mother’s mtDNA is combined with the donor’s mitochondrial genome, it only constitutes about 0.1% of DNA, so it should not be considered a third parent [161].

### 4.2. Resveratrol

Resveratrol (RSV) is a natural polyphenol that can be found in various plants, including grapes and peanuts, as well as in wine and tea [162,163]. Plants synthesise RSV in response to stressful conditions, including extreme temperatures, mechanical damage, ultraviolet irradiation, and pathogen attacks [162,164]. It is considered a phytoestrogen because it shares structural and functional similarities with oestrogen [165]. Resveratrol is well documented for its diverse range of properties, including anti-ageing, antioxidant, anti-inflammatory, insulin sensitising, cardioprotective, vasodilatory, and anticancer effects [164,165,166]. Furthermore, studies have highlighted its efficacy in improving health and mitigating chronic diseases, including neurodegenerative conditions [167].

The function of resveratrol is based on its interaction with various molecular targets, including active enzyme sites and proteins such as tubulin, protein kinase C alpha (PKCα), phosphodiesterase-4D, human oral cancer cell line proteins, PKCα, and lysine-specific demethylase 1 [168]. Within cells, resveratrol exerts its effects by influencing signalling pathways, including the nuclear factor-κB (NF-κB) pathway. NF-κB is a critical regulator of immune responses, cell survival, and ovarian cell functions, encompassing processes such as proliferation, apoptosis, and the release of steroid hormones [168,169]. Additionally, resveratrol modulates pathways related to inflammatory prostaglandins and cytokines, as well as antioxidant enzymes, which are key players in apoptosis regulation, mitochondrial biogenesis, gluconeogenesis, and lipid metabolism. Furthermore, the impact on DNA methylation has been demonstrated, implicating its potential role in epigenetic control over oxidative, metabolic, and tumorigenic processes [170].

Resveratrol acts as a potent activator of the silent information regulator 2 type 1 (SIRT1) [167,171]. Its role in the regulation of ovarian function and the modulation of steroidogenesis through sirtuins has been well established [172]. Sirtuins belong to the nicotinamide adenine dinucleotide-dependent deacetylase protein family and are recognised for their involvement in various cellular processes, including apoptosis, DNA repair, lipid metabolism, redox homeostasis, and ovarian ageing [162,173]. In mammals, seven members of the sirtuins family are distinguished [174]. SIRT1 has been proven to be expressed in animal ovaries, mammal embryos, and oocytes [171]. Moreover, it supervises oocyte maturation and is also involved in the regulation of granulosa cell apoptosis during follicular atresia, contributing to the extension of the ovarian lifespan [171,175].

Recent research has proven evidence that SIRT1 stimulates mitochondrial biogenesis while also regulating mitochondrial functionality [171]. Resveratrol improves mitochondrial function through SIRT1 activation and activates sirtuins, which play a role in anti-ageing processes [175,176]. SIRT1 activation leads to SIRT1 deacetylation of SIRT1 and the subsequent activation of the peroxisome proliferator-activated receptor-gamma coactivator (PGC-1α,) which in turn regulates mitochondrial biogenesis [177]. PGC-1α serves as a critical transcriptional coactivator that plays a central role in regulating energy metabolism [178]. The presence of stress triggers the activation of SIRT1 and its associated molecular targets, including the kappa light chain enhancer of activated B cells (NF-kB), tumour protein p53, forkhead box (FoxO), PGC-1α, liver X receptor, nibrin (NBS1), and hypoxia-inducible factor 2α (HIF-2α) [162,171]. As the most potent natural SIRT1 ligand, resveratrol emerges as a significant contributor to the maintenance of energy equilibrium, the facilitation of gene regulation, and the protection of genome stability, all of which are integral to cell viability [171]. In this capacity, resveratrol plays a central role in the governance of a multitude of processes that span energy regulation, gene expression, genomic integrity, and cell survival [162]. Furthermore, resveratrol exhibits therapeutic potential in female reproduction, with multiple studies highlighting its beneficial effects in conditions such as endometriosis, breast cancer, and cervical cancer [179,180,181,182]. It has been found to promote apoptosis and autophagy while inhibiting the migration and invasion of human cervical cancer cells [180]. RSV has also demonstrated protective effects against ovarian cancer in mouse ovaries by increasing glucose uptake and exhibiting anti-tumour properties. A study conducted by Bezerra et al. [183] on sheep ovaries provided evidence that resveratrol stimulates granulosa cell proliferation and activates primordial follicles [183]. Furthermore, research on a rat model of premature ovarian failure underscores the antioxidant properties of resveratrol on granulosa cells, attributed to its modulation of the PI3K/Akt/mTOR signalling pathway, a critical regulator of oocyte growth, primordial follicle development, and proliferation of granulosa cells [184]. In a study conducted by Nishigaki et al. [173] on a human ovarian granulosa-like tumour cell line (KGN) exposed to cobalt chloride (CoCl_2_) as a hypoxic factor at concentrations of 10 µmol/L and 100 µmol/L, it was shown that resveratrol supplementation at concentrations of 10, 25, and 50 μmol/L directly influenced the SIRT1/PGC-1α pathway. The study observed that CoCl_2_ reduced the expression of SIRT1 and the number of mtDNA copies, while the addition of resveratrol counteracted this negative effect [178].

Research has shown its ability to stimulate mitochondrial activity, leading to increased ATP production and improved cell viability in human granulosa cells [185,186]. RSV treatment has been shown to enhance autophagy in bovine granulosa cells [174]. Furthermore, the addition of resveratrol to the culture medium during in vitro oocyte culture has been reported to have beneficial effects on the oocytes, improving the ATP content and the fertilisation process [174]. Sugiyama et al. [174], in complexes of bovine oocyte and granulosa cells obtained from aged cows (>120 months of age), demonstrated that resveratrol increased SIRT1 expression and induced autophagy in both granulosa cells and oocytes. Furthermore, in in vitro cultures, RSV was found to have a significant impact on mitochondrial DNA copy numbers in bovine oocytes, leading to higher ATP content [174]. Similar results were obtained in the study conducted on aged mouse oocytes [176]. The authors indicated that long-term RSV treatment (22 weeks) helped prevent the degradation of oocyte quality in mice, as well as restored implantation rates and live offspring production [176]. Furthermore, this natural polyphenol maintained mitochondrial activity in female gametes, even during short-term supplementation (7 days) [176]. Studies indicated that resveratrol supplementation can help prevent ageing and the decline in its quality [174,176]. Furthermore, resveratrol has exhibited a favourable impact on the mitochondria of vitrified oocytes. Vitrification usually results in decreased ATP levels and increased reactive oxygen species in bovine, mouse, and porcine oocytes [187]. These changes can have adverse effects on electron transport, calcium regulation, DNA integrity, and mitochondrial morphology [187]. However, supplementation of the culture medium with resveratrol (1 µM) during the post-vitrification warming process has been shown to enhance mitochondrial function. Subsequently, this improvement leads to increased oocyte survival and maturation, along with a reduction in apoptosis rates and reactive oxygen species levels [187]. Furthermore, additional research has indicated that intraperitoneal administration of resveratrol at a dose of 20 mg/kg body weight enhances embryonic development during the superovulation process in mice [186]. This improvement is attributed to enhanced oocyte mitochondrial function, elevated gene expression associated with mitochondrial biogenesis, and an increased blastocyst formation rate. Specifically, the test group receiving resveratrol demonstrated a 20% higher rate of reaching the blastocyst stage (61.67%) compared to the control group without resveratrol supplementation [186].

### 4.3. Leonurine

Leonurine (also known as SCM-198 or LEO) is a natural alkaloid found in the leaves of *Leonurus japonicus* Houtt [188]. This compound exhibits a robust capacity to counteract free radicals, quench reactive oxygen species, and elevate the levels of crucial enzymes such as superoxide dismutase, catalase, and glutathione peroxidase [189]. LEO is associated with anti-inflammatory, antioxidant, anti-platelet aggregation, uterine stimulation, anti-tumour, and cardiovascular protective properties [190,191]. Furthermore, it has the potential to improve mitochondrial dysfunction [192].

The protective effects of LEO on ovarian health were demonstrated in a study conducted by Chi et al. [193]. The research involved mice divided into four experimental groups, along with a control group. The first group received cyclophosphamide (CTX) at a dosage of 150 mg/kg/week, administered intraperitoneally in physiological saline daily. The subsequent three groups received CTX along with intraperitoneal injections of leonurine hydrochloride at various concentrations (7.5 mg/kg, 15 mg/kg, and 30 mg/kg) for a treatment period of 28 days. After this treatment, the mice were mated with males and sacrificed on the 14th day of pregnancy. CTX administration was found to induce premature ovarian insufficiency (POI). This induction led to a significant reduction in ovarian weight, although their relative weight returned to normal with LEO doses of 15 and 30 mg/kg. These findings support the notion that leonurine can ameliorate the condition of POI mice, positively influencing implantation and the number of live foetuses [193]. Furthermore, the study’s authors demonstrated that LEO has a regulatory impact on the pyroptosis pathway, which is implicated in the development of POI. LEO was shown to modulate the expression levels of the NLRP3, ASC, cleaved caspase-1, and GSDMD proteins. Furthermore, it reduced the levels of interleukin18 in the serum, as compared to the group with POI [193]. In another study conducted by Zheng et al. [188], the impact of LEO on bovine oocytes and embryos was explored. To assess its influence on oocyte development potential, various concentrations of LEO (0 µM, 20 µM, 40 µM, and 80 µM) were introduced into the culture medium. Remarkably, enhanced development was observed even at the lowest alkaloid concentration, surpassing the control sample. LEO treatment also resulted in a heightened rate of blastocyst formation. Additionally, compared to the control group, lower levels of reactive oxygen species and glutathione were detected in bovine oocytes exposed to leonurine [188]. Furthermore, it was demonstrated that LEO—beyond its capacity to reduce ROS—appears to engage in lipid synthesis and metabolism. This involvement influences the availability and absorption of fatty acids, phospholipids, and triglycerides by oocytes [188].

Although there are differences in the experimental conditions of the studies described above (the experiment of Chi et al. [193] focused on the protective effect of LEO against toxic damage to the ovaries in mice, while the study of Zheng et al. [188] analysed the direct effect of LEO on the quality and development of oocytes and bovine embryos), both teams proved that LEO has a beneficial effect on the health and functioning of germ cells, both in the animal model (mice) and in in vitro studies on bovine oocytes. Both experiments indicate that LEO protects oocytes and ovaries from negative factors, such as oxidative stress or toxic substances (e.g., cyclophosphamide). It improves the functioning of the reproductive system, increases the chances of successful embryo implantation and foetal development, and affects metabolic processes, regulating the levels of ROS, lipids, and inflammatory markers.

The influence of leonurine was assessed on porcine ovaries and oocytes [192]. The research revealed that LEO, administered in various concentrations (0 μM, 20 μM, 40 μM, and 60 μM), exerts a notable effect on early embryo development. In particular, the concentration of 40 μM LEO demonstrated the highest efficacy in enhancing porcine blastocyst formation (72.22% ± 3.78%) compared to other concentrations (47.43% ± 1.01% for 0%, 56.47% ± 2.53% for 20%, and 59.54% ± 3.40% for 60%) [192].

Leonurine has the ability to suppress intracellular reactive oxygen species and mitigate mitochondrial dysfunction [194]. It facilitates Akt phosphorylation, which subsequently inhibits cytochrome c and the secretion of inflammatory cytokine secretion and down-regulates the Bcl-2/Bax ratio [194]. Leonurine enhances the levels of the anti-apoptotic marker Bcl-2 while reducing those of the apoptotic marker Bax [195]. This multifaceted action leads to the attenuation of caspase activation and results in anti-apoptotic, antioxidant, and anti-inflammatory effects [194].

The results of these studies suggest that LEO may be a potential agent that supports fertility and ovarian health, which may be important for the design of future therapies in reproductive medicine.

### 4.4. Melatonin

Melatonin (5-methoxy-N-acetyltryptamine) is a hormone secreted by the pineal gland, but also by other tissues such as the retina, placenta, and ovary [196]. Additionally, the follicular granulosa cells in oocytes are capable of synthesising melatonin [197]. This is a multifunctional substance that includes antioxidant and anti-inflammatory properties [198]. It can contribute to the modulation of mitochondrial functions according to its antioxidant properties and reduce free radical levels [197]. Melatonin is involved in regulating ovarian function, which encompasses oocyte maturation, the mitigation of oxidative harm in granulosa cells, and the safeguarding of granulosa cells from undergoing apoptosis [173]. It has been shown that melatonin levels in the blood and in the follicular fluid decrease with maternal ageing [196,197].

Research conducted on both young (6–8 weeks old) and aged (50–60 weeks old) mouse ovaries aimed to investigate the impact of melatonin on communication between granulosa cells and oocytes [197]. In this study, one group of mice received melatonin treatment (30 mg/kg body weight) for a duration of 28 days, while the second group served as a control and received water. The research revealed that oral melatonin supplementation in older mice led to a notable increase in the number of transzonal projections (TZP) [197]. These TZPs are specialised filopodia that envelop cumulus granulosa cells and extend to reach oocytes, facilitating communication between oocytes and somatic cells. Furthermore, the administration of melatonin was found to enhance the morphology of cumulus-oocyte complexes, which is particularly important since COC quality tends to deteriorate with age [197].

Furthermore, the study included an examination of the impact of reducing reactive oxygen species in in vitro oocyte culture [197]. Fluorescence analysis confirmed that melatonin supplementation effectively curtailed ROS accumulation in aged mouse oocytes. Melatonin was further shown to enhance the activity of glucose-6-phosphate dehydrogenase (G6PDH) and restore favourable ratios of NADPH/NADP+ and GSH/GSSG in older COCs. This action reduces the excessive buildup of ROS and serves as a protective measure against oxidative damage in oocytes [197].

A similar study examined the impact of melatonin on the in vitro maturation of mouse oocytes [196]. To explore this, the oocytes were cultured in a medium enriched with varying concentrations of melatonin (10^−4^, 10^−6^, and 10^−8^ M/L). The findings revealed that the addition of melatonin substantially enhanced the fertilisation potential of ageing oocytes, resulting in an average improvement of 20–30% compared to the control group that did not receive melatonin supplementation [196]. A detailed analysis conducted by the researchers indicated that melatonin supplementation reduced the proportion of oocytes exhibiting heterogeneous mitochondria compared to the control group (melatonin: 32.71 ± 0.81%, n = 72 vs. control: 43.06 ± 0.69%, n = 85, *p* < 0.01) [196]. The study demonstrated that melatonin plays a constructive role in both oocyte maturation and early embryonic development. Melatonin’s mechanism of action involves the regulation of oxidative stress through its influence on the expression of key genes like *SIRT1* and *SIRT3*, alongside other antioxidant genes, including *SOD1*, *SOD2*, and *GPX4* [196].

Xu et al. [173] conducted an experiment involving bovine granulosa cells exposed to varying concentrations of hydrogen peroxide (H_2_O_2_) (ranging from 0 µM to 1000 µM) for a duration of 4 h. Subsequently, these cells were treated with different concentrations of melatonin (ranging from 0 µM to 10 µM). The results indicated that 400 µM H_2_O_2_ reduced cell viability by approximately 50% [173]. However, when melatonin was introduced, it demonstrated a positive effect on cell viability. Furthermore, melatonin mitigated H_2_O_2_-induced depolarisation of the mitochondrial membrane in ovarian granulosa cells. In addition, melatonin was observed to inhibit apoptosis in bovine ovarian granulosa cells. This effect was attributed to melatonin’s ability to induce mitophagy by activating SIRT1 expression, which subsequently led to the deacetylation of FoxO1 [173].

### 4.5. L-Carnitine

L-carnitine (LC) is a small, hydrophilic molecule initially isolated from cattle muscles [199]. It serves a crucial role in lipid metabolism and mitochondrial function [200]. LC is essential for ATP production and concurrently reduces oxidative stress within cells [201]. It facilitates the translocation of acetyl groups from the cytoplasm to the mitochondrion and regulates the activity of mitochondrial enzymes [202,203]. Additionally, it contributes to the elimination of xenobiotics from cells [204]. LC possesses the capacity to transport long-chain fatty acids across the inner mitochondrial membrane. These fatty acids then undergo β-oxidation, resulting in the formation of acetyl fragments in the form of acetyl-coenzyme A. Subsequently, these acetyl-CoA molecules enter the Krebs cycle, participating in ATP production [204]. L-carnitine plays a crucial role in maintaining optimal levels of acetyl-CoA, thereby ensuring the efficiency of the pyruvate oxidation and glycolysis reactions [204].

The impact of L-carnitine on the growth of buffalo oocytes was investigated by Modak et al. [205]. Buffalo oocytes were cultured in a medium supplemented with varying concentrations of L-carnitine: 0, 1.25, 1.875, and 2.50 mM. The addition of 1.875 and 2.5 mM LC significantly increased the diameters compared to the lower concentration of 1.25 mM and the control group (0 mM). On the sixth day of culture, oocytes supplemented with 2.5 mM LC reached an average diameter of 118.8 ± 0.5 μm, while those treated with 1.875 mM LC measured 115.8 ± 0.4 μm. Moreover, on the third day of culture, the percentage of degenerated oocytes was lower for concentrations of 0, 1.25, 1.875, and 2.50 mM, with corresponding values of 45%, 40%, 30%, and 7.5%, respectively. Conversely, for oocytes treated with 2.50 mM L-carnitine, the degeneration rate on the sixth day was 10%, while for the 1.25 mM concentration, a considerable 45% of oocytes degenerated [205].

Exposure to LC has been shown to reduce apoptosis and elevate glutathione levels in porcine oocytes. In the in vitro culture of bovine oocytes, an increased frequency of mature oocytes with scattered mitochondria was observed following LC treatment [201]. Studies conducted on in vitro cultured lamb oocytes treated with acetyl-L-carnitine (ALC), a natural derivative of L-carnitine easily converted into L-carnitine, demonstrated its influence on the expansion of cumulus cells and a twofold increase in the number of blastocysts compared to the control without carnitine supplementation [201]. Another study involving porcine oocytes matured in vitro with medium supplemented with varying concentrations of L-carnitine (0, 0.25, 0.5, 1, and 2 mg/mL) revealed that the addition of 0.5 mg/mL L-carnitine to in vitro maturation (IVM) media reduced ROS levels in oocytes and embryos compared to the control group [199]. Additionally, LC exhibited a beneficial effect on nuclear maturation, thus improving the developmental competence of porcine oocytes [199]. In a subsequent investigation by Catandi et al. [206], the impact of L-carnitine (3 mM) on maturation in vitro was explored. The results demonstrated that oocytes exposed to LC during IVM exhibited reduced mitochondrial ROS levels compared to the control group without LC supplementation [206]. It should be noted that both oocytes and cumulus cells lack the capacity to synthesise L-carnitine, and the addition of LC to the culture medium was correlated with increased mitochondrial membrane potential and reduced ROS levels [206].

### 4.6. Coenzyme Q10

Coenzyme Q10 (CoQ10) is a natural compound present in biological membranes and mitochondria, and its synthesis takes place in all human cells [207,208]. This molecule bears structural similarities to vitamin K and boasts potent antioxidant properties [209]. CoQ10 plays an essential role in antioxidant mechanisms, gene regulation, energy metabolism, and anti-inflammatory processes [210,211]. Notably, CoQ10 plays a pivotal role in the mitochondrial electron transport chain, facilitating the generation of cellular energy [212]. In mammals, the de novo synthesis of Coenzyme Q10 is involved in the expression of the *PDSS-2* and *COQ-6* genes [209]. Disturbing the expression of these two genes in the ovary has been shown to disrupt the functionality of oocytes, ovarian follicles, and mitochondria in both human and mouse models [209]. With age, the levels of CoQ10 in the serum decrease, but supplementation of the culture medium restores the functions of pig oocytes, which tend to exhibit lower quality with age [213].

Yang et al. [214] conducted a study to explore the impact of CoQ10 at different concentrations (25, 50, and 100 µM) on the maturation of pig oocytes. Their findings revealed that the 50 µM concentration of CoQ10 exhibited the superior potential to promote nuclear maturation of porcine oocytes, regardless of whether they originated from small or large antral follicles [214]. Furthermore, this study observed that oocytes treated with 50 µM CoQ10 showed a significantly heightened mitochondrial membrane potential (1.32) compared to the control group (1.00). Notably, the addition of 50 µM coenzyme Q10 led to a twofold increase in ATP production [214].

Additional research explored the influence of CoQ10 on oocyte in vitro maturation, mitochondrial characteristics, and ROS levels [215]. Lamb oocytes were obtained for this purpose and cultured in vitro with media supplemented with CoQ10 at concentrations of 0, 15, 30, and 50 µM. The group treated with 30 µM coenzyme exhibited the highest blastocyst formation rate (42.36 ± 3.99) compared to the control group (29.43 ± 1.83) [215]. Furthermore, the mean value of the relative mitochondrial mass was significantly elevated in the group treated with 30 µM CoQ10 (18.81 ± 0.26) compared to the control group (17.46 ± 0.46). Additionally, this group demonstrated an increase in mitochondrial inner membrane potential and nearly half the ROS content of the control group [215].

Mitoquinone, another mitochondria-targeted antioxidant, mirrors the effects of CoQ10 [216]. This compound can accumulate within mitochondria. The current literature emphasises its role in promoting in vitro oocyte maturation by preserving mitochondrial homeostasis. Investigations on a human ovarian granulosa cell line (HGL5) supplemented with 10 nM MitoQ, as well as studies on mouse oocytes (over 40 weeks old) treated daily with 10 nM MitoQ, revealed its ability to safeguard HGL5 cells from oxidative stress-induced damage and inhibit mitochondrial ROS production. These studies further illustrated that MitoQ supplementation enhances mitochondrial function in ageing oocytes [216].

**Figure 4:**
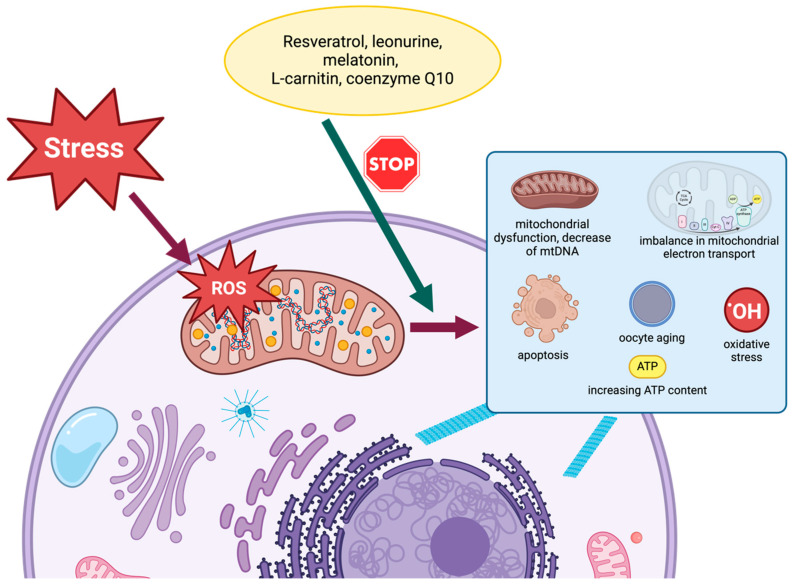
The effect of different substances on mitochondrial functions (Created with Biorender.com accessed on 28 January 2025).

The effect of different substances on the possibility of mitochondrial regeneration is shown in Figure 4, and a summary of the properties of the described substances is provided in Table 1.

## 5. Conclusions

Mitochondria play an indispensable role as energy and metabolic centres of cells, significantly influencing female reproductive capacity. Their proper functioning is crucial for oogenesis, fertilisation, and embryonic development, while mitochondrial dysfunction is closely associated with reproductive disorders and diseases. Understanding the processes that govern mitochondrial activity and their interaction with oocyte and embryo quality has broad implications for improving reproductive outcomes. Studies emphasise the importance of targeting mitochondrial processes through interventions such as antioxidant supplementation, mitochondrial replacement therapy, and compounds such as resveratrol, melatonin, or coenzyme Q10. These strategies not only improve mitochondrial function but also increase oocyte quality, embryonic development, and overall reproductive success. Further research is needed to develop improved, targeted approaches to addressing mitochondrial dysfunction in reproductive medicine.

## Figures and Tables

**Table 1:** Summary of the properties of various substances and their impact on ovarian health and oocyte quality.

Sunstance	Source	Functions	Effects on Oocytes, and Ovarian Health
Resveratrol	Grapes, peanuts, wine, tea	- Antioxidant, anti-inflammatory, anti-ageing, insulin sensitizing, cardioprotective, and anticancer effects	- Improves mitochondrial function and ATP production in oocytes. - Protects oocyte quality and function in aging ovaries. - Enhances fertilization and blastocyst formation rates. - Protects against oxidative stress, improves oocyte maturation.
Leonurine	*Leonurus japonicus* (Herb)	- Antioxidant, anti-inflammatory, anti-tumour, anti-platelet aggregation, uterine stimulation, and mitochondrial dysfunction improvement	- Reduces ROS levels in oocytes and embryos. - Enhances blastocyst formation in oocytes. - Improves mitochondrial function and fertility in animal models. - Reduces apoptotic markers in ovaries and enhances follicle health.
Melatonin	Pineal gland, retina, ovary	- Antioxidant and anti-inflammatory and regulates circadian rhythms, mitochondrial function, and ROS levels	- Increases oocyte maturation and fertilization potential. - Protects oocytes from oxidative stress. - Enhances cumulus-oocyte complex quality. - Improves mitochondrial function in aged oocytes.
L-carnitine	Animal muscle tissues	- Facilitates lipid metabolism, mitochondrial function, and ATP production and reduces oxidative stress	- Reduces apoptosis and enhances mitochondrial function in oocytes. - Increases oocyte diameter and developmental potential. - Reduces ROS levels and improves maturation rates in various species (buffalo, porcine, and lamb).
Coenzyme Q10	Mitochondria, biological membranes	- Antioxidant, mitochondrial energy production, gene regulation, and anti-inflammatory effects.	- Promotes nuclear maturation and mitochondrial health in oocytes. - Enhances mitochondrial membrane potential and ATP production. - Reduces ROS in oocytes, improving their quality and developmental potential.

## Abbreviations

2-APB	2-aminoethoxydiphenyl borate
Acetyl-CoA	Acetyl Coenzyme A
ALC	acetyl-L-carnitine
AMPK	AMP-activated protein kinase
ART	assisted reproductive technologies
ATP	adenosine triphosphate
C	cytochrome C
CABL1	critical calcium-binding protein
*Calb1*	calbindin 1 gene
CL	cardiolipin
CoCl_2_	cobalt chloride
COCs	cumulus-oocyte complexes
CoQ	Coenzyme Q10
CoQ10	coenzyme Q10
COX	cytochrome c oxidase
CTX	cyclophosphamide
DCA	dichloroacetate
DOR	diminished ovarian reserve
ER	endoplasmic reticulum
ETC	electron transport chain
FA	fatty acid
FASN	fatty acid synthase
FoxO	forkhead box
G6PDH	glucose-6-phosphate dehydrogenase
GAPDH	glyceraldehyde-3-phosphate dehydrogenase
GLUT	glucose transporter
GPx	glutathione peroxidase
GR	glutathione *reductase*
GV	germinal vesicle
H_2_O_2_	hydrogen peroxide
HIF-2α	hypoxia-inducible factor 2 alpha
HIF1α	hypoxia-inducible factor 1α
HS	heat stress
HSP	heat shock protein
IA	iodoacetate
IMM	inner mitochondrial membrane
IMS	mitochondrial intermembrane space
IP3R	inositol trisphosphate receptor
IVF	in vitro fertilisation
IVM	in vitro maturation
LC	L- carnitine
MCT	monocarboxylate transporter
MCU	mitochondrial calcium uniporter
Mfn 1	Mitofusin 1
Mfn 2	Mitofusin 2
MPC	mitochondrial pyruvate carrier
*MRT*	mitochondrial replacement therapy
mtDNA	mitochondrial DNA
mTOR	mechanistic target of rapamycin
NAD+	nicotinamide adenine dinucleotide
NADPH	nicotinamide adenine dinucleotide phosphate
NAM	nicotinamide
NBS1	nibrin
NDs	neurodegenerative diseases
NF-κB	nuclear factor-κB
OAA	oxaloacetate
OMM	outer mitochondrial membrane
OXPHOS	oxidative phosphorylation
PAHs	polycyclic aromatic hydrocarbons
PBE	polar body extrusion
PDH	Pyruvate dehydrogenase
PDK	pyruvate dehydrogenase kinase
PGC-1α	proliferator-activated receptor-gamma coactivator
PKCα	protein kinase C alpha
POA	premature ovarian ageing
POI	premature ovarian insufficiency
PPP	pentose phosphate pathway
Q10	coenzyme Q10
ROS	reactive oxygen species
RSV	resveratrol
RyR	ryanodine receptor
SCM-198, LEO	leonurine
SIRT	sirtuin, silent information regulator
SOD	superoxide dismutase
SOD1	superoxide dismutase 3
SOD2	superoxide dismutase 2
TAG	triacylglyceride
TCA	tricarboxylic acid cycle
TG	thapsigargin
TZP	transzonal projections
VDAC	voltage-dependent anion channel

## References

[1] Moretti-Horten D.N., Peselj C., Taskin A.A., Myketin L., Schulte U., Einsle O., Drepper F., Luzarowski M., Vögtle F.-N. (2024). Synchronized assembly of the oxidative phosphorylation system controls mitochondrial respiration in yeast. Dev. Cell.

[2] Masuda D., Nakanishi I., Ohkubo K., Ito H., Matsumoto K., Ichikawa H., Chatatikun M., Klangbud W.K., Kotepui M., Imai M. (2024). Mitochondria Play Essential Roles in Intracellular Protection against Oxidative Stress—Which Molecules among the ROS Generated in the Mitochondria Can Escape the Mitochondria and Contribute to Signal Activation in Cytosol?. Biomolecules.

[3] Schiaffarino O., Valdivieso González D., García-Pérez I.M., Peñalva D.A., Almendro-Vedia V.G., Natale P., López-Montero I. (2022). Mitochondrial membrane models built from native lipid extracts: Interfacial and transport properties. Front. Mol. Biosci..

[4] Dong J., Chen L., Ye F., Tang J., Liu B., Lin J., Zhou P.-H., Lu B., Wu M., Lu J.-H. (2024). Mic19 depletion impairs endoplasmic reticulum-mitochondrial contacts and mitochondrial lipid metabolism and triggers liver disease. Nat. Commun..

[5] Decker S.T., Funai K. (2024). Mitochondrial membrane lipids in the regulation of bioenergetic flux. Cell Metab..

[6] Koma R., Shibaguchi T., Pérez López C., Oka T., Jue T., Takakura H., Masuda K. (2021). Localization of myoglobin in mitochondria: Implication in regulation of mitochondrial respiration in rat skeletal muscle. Physiol. Rep..

[7] Chen W., Zhao H., Li Y. (2023). Mitochondrial dynamics in health and disease: Mechanisms and potential targets. Signal Transduct. Target. Ther..

[8] Jin S., He Y., Feng C., Yuan J., Guo Y., Guo Z., Wang X. (2025). Cellular Discrepancy of Platinum Complexes in Interfering with Mitochondrial DNA. ACS Cent. Sci..

[9] Yildirim R.M., Seli E. (2024). The role of mitochondrial dynamics in oocyte and early embryo development. Semin. Cell Dev. Biol..

[10] Li L.-F., Yu J., Li R., Li S.-S., Huang J.-Y., Wang M.-D., Jiang L.-N., Xu J.-H., Wang Z. (2024). Apoptosis, Mitochondrial Autophagy, Fission, and Fusion Maintain Mitochondrial Homeostasis in Mouse Liver Under Tail Suspension Conditions. Int. J. Mol. Sci..

[11] Dong F., Zhu M., Zheng F., Fu C. (2022). Mitochondrial fusion and fission are required for proper mitochondrial function and cell proliferation in fission yeast. FEBS J..

[12] Dubois M., Boulghobra D., Rochebloine G., Pallot F., Yehya M., Bornard I., Gayrard S., Coste F., Walther G., Meyer G. (2024). Hyperglycemia triggers RyR2-dependent alterations of mitochondrial calcium homeostasis in response to cardiac ischemia-reperfusion: Key role of DRP1 activation. Redox Biol..

[13] Cheng S., Altmeppen G., So C., Welp L.M., Penir S., Ruhwedel T., Menelaou K., Harasimov K., Stützer A., Blayney M. (2022). Mammalian oocytes store mRNAs in a mitochondria-associated membraneless compartment. Science.

[14] Chuang T.-H., Chou H.-H., Kuan C.-S., Liu S.-C., Kao C.-W., Wu Y.-H., Lai H.-H., Hsieh C.-L., Liang Y.-T., Chen C.-Y. (2024). Dependency of mitochondrial quantity on blastocyst timeline obscures its actual effect to pregnancy outcomes. Front. Endocrinol..

[15] Hao X., Zhao J., Rodriguez-Wallberg K.A. (2024). Comprehensive atlas of mitochondrial distribution and dynamics during oocyte maturation in mouse models. Biomark. Res..

[16] Contreras-Solís I., Catalá M., Soto-Heras S., Roura M., Paramio M.T., Izquierdo D. (2021). Effect of follicle size on hormonal status of follicular fluid, oocyte ATP content, and in vitro embryo production in prepubertal sheep. Domest. Anim. Endocrinol..

[17] Sobek A., Tkadlec E., Klaskova E., Prochazka M. (2021). Cytoplasmic Transfer Improves Human Egg Fertilization and Embryo Quality: An Evaluation of Sibling Oocytes in Women with Low Oocyte Quality. Reprod. Sci..

[18] Ge H., Tollner T.L., Hu Z., Dai M., Li X., Guan H., Shan D., Zhang X., Lv J., Huang C. (2012). The importance of mitochondrial metabolic activity and mitochondrial DNA replication during oocyte maturation in vitro on oocyte quality and subsequent embryo developmental competence. Mol. Reprod. Dev..

[19] Cimadomo D., Fabozzi G., Vaiarelli A., Ubaldi N., Ubaldi F.M., Rienzi L. (2018). Impact of Maternal Age on Oocyte and Embryo Competence. Front. Endocrinol..

[20] Van Blerkom J. (2011). Mitochondrial function in the human oocyte and embryo and their role in developmental competence. Mitochondrion.

[21] Dumollard R., Duchen M., Carroll J. (2007). The Role of Mitochondrial Function in the Oocyte and Embryo. Current Topics in Developmental Biology.

[22] Yildirim R.M., Seli E. (2024). Mitochondria as therapeutic targets in assisted reproduction. Human Reprod..

[23] Liang D., Zhu L., Zhu Y., Huang M., Lin Y., Li H., Hu P., Zhang J., Shen B., Xu Z. (2024). A PCR-independent approach for mtDNA enrichment and next-generation sequencing: Comprehensive evaluation and clinical application. J. Transl. Med..

[24] Koller A., Fazzini F., Lamina C., Rantner B., Kollerits B., Stadler M., Klein-Weigel P., Fraedrich G., Kronenberg F. (2020). Mitochondrial DNA copy number is associated with all-cause mortality and cardiovascular events in patients with peripheral arterial disease. J. Intern. Med..

[25] Colnaghi M., Pomiankowski A., Lane N. (2021). The need for high-quality oocyte mitochondria at extreme ploidy dictates mammalian germline development. eLife.

[26] Khan S.A., Reed L., Schoolcraft W.B., Yuan Y., Krisher R.L. (2023). Control of mitochondrial integrity influences oocyte quality during reproductive aging. Mol. Human Reprod..

[27] Lu X., Liu Y., Xu J., Cao X., Zhang D., Liu M., Liu S., Dong X., Shi H. (2022). Mitochondrial dysfunction in cumulus cells is related to decreased reproductive capacity in advanced-age women. Fertil. Steril..

[28] Lu L., Wang T., Liu A., Ye H. (2025). A Single-Cell Atlas of Crab Ovary Provides New Insights into Oogenesis in Crustaceans. Adv. Sci..

[29] Chiaratti M.R. (2021). Uncovering the important role of mitochondrial dynamics in oogenesis: Impact on fertility and metabolic disorder transmission. Biophys. Rev..

[30] Shan L.-Y., Tian Y., Liu W.-X., Fan H.-T., Li F.-G., Liu W.-J., Li A., Shen W., Sun Q.-Y., Liu Y.-B. (2023). LSM14B controls oocyte mRNA storage and stability to ensure female fertility. Cell. Mol. Life Sci..

[31] Li H., Zhao H., Yang C., Su R., Long M., Liu J., Shi L., Xue Y., Su Y. (2023). LSM14B is an Oocyte-Specific RNA-Binding Protein Indispensable for Maternal mRNA Metabolism and Oocyte Development in Mice. Adv. Sci..

[32] Guo X., Jiao L., Yi Y., Zhang H., Liu Y., Wang Z., Sun S. (2024). NAMPT regulates mitochondria function and lipid metabolism during porcine oocyte maturation. J. Cell. Physiol..

[33] Lee I.-W., Adhikari D., Carroll J. (2022). Miro1 depletion disrupts spatial distribution of mitochondria and leads to oocyte maturation defects. Front. Cell Dev. Biol..

[34] Deng K., Du D., Fan D., Pei Z., Zhang S., Xu C. (2023). Growth Hormone Promotes Oocyte Maturation In Vitro by Protecting Mitochondrial Function and Reducing Apoptosis. Reprod. Sci..

[35] Satouh Y., Sato K. (2023). Reorganization, specialization, and degradation of oocyte maternal components for early development. Reprod. Med. Biol..

[36] Marei W.F.A., Mohey-Elsaeed O., Pintelon I., Leroy J.L.M.R. (2024). Risks of using mitoquinone during in vitro maturation and its potential protective effects against lipotoxicity-induced oocyte mitochondrial stress. J. Assist. Reprod. Genet..

[37] Ju W., Zhao Y., Yu Y., Zhao S., Xiang S., Lian F. (2024). Mechanisms of mitochondrial dysfunction in ovarian aging and potential interventions. Front. Endocrinol..

[38] Li X.-Q., Wang Y., Yang S.-J., Liu Y., Ma X., Liu L., Li S.-H., Niu D., Duan X. (2022). Melatonin protects against maternal diabetes-associated meiotic defects by maintaining mitochondrial function. Free Radic. Biol. Med..

[39] Wang Q., Ratchford A.M., Chi M.M.-Y., Schoeller E., Frolova A., Schedl T., Moley K.H. (2009). Maternal Diabetes Causes Mitochondrial Dysfunction and Meiotic Defects in Murine Oocytes. Mol. Endocrinol..

[40] Hou X., Zhu S., Zhang H., Li C., Qiu D., Ge J., Guo X., Wang Q. (2019). Mitofusin1 in oocyte is essential for female fertility. Redox Biol..

[41] Li X., Straub J., Medeiros T.C., Mehra C., Den Brave F., Peker E., Atanassov I., Stillger K., Michaelis J.B., Burbridge E. (2022). Mitochondria shed their outer membrane in response to infection-induced stress. Science.

[42] Inagaki S., Suzuki Y., Kawasaki K., Kondo R., Imaizumi Y., Yamamura H. (2023). Mitofusin 1 and 2 differentially regulate mitochondrial function underlying Ca^2+^ signaling and proliferation in rat aortic smooth muscle cells. Biochem. Biophys. Res. Commun..

[43] Ning Y., Cai Y., Dai Y., Li F., Mo S., Werz O., Chen X. (2021). Mitochondrial Fusion Mediated by Mitofusin 1 Regulates Macrophage Mycobactericidal Activity by Enhancing Autophagy. Infect. Immun..

[44] Rodríguez-Nuevo A., Torres-Sanchez A., Duran J.M., De Guirior C., Martínez-Zamora M.A., Böke E. (2022). Oocytes maintain ROS-free mitochondrial metabolism by suppressing complex I. Nature.

[45] Onukwufor J.O., Farooqi M.A., Vodičková A., Koren S.A., Baldzizhar A., Berry B.J., Beutner G., Porter G.A., Belousov V., Grossfield A. (2022). A reversible mitochondrial complex I thiol switch mediates hypoxic avoidance behavior in *C. elegans*. Nat. Commun..

[46] Elías-López A.L., Vázquez-Mena O., Sferruzzi-Perri A.N. (2023). Mitochondrial dysfunction in the offspring of obese mothers and it’s transmission through damaged oocyte mitochondria: Integration of mechanisms. Biochim. Biophys. Acta (BBA)-Mol. Basis Dis..

[47] Wang F., Meng T.-G., Li J., Hou Y., Luo S.-M., Schatten H., Sun Q.-Y., Ou X.-H. (2020). Mitochondrial Ca^2+^ Is Related to Mitochondrial Activity and Dynamic Events in Mouse Oocytes. Front. Cell Dev. Biol..

[48] Cheng J., Wang X., Luo C., Mao X., Qin J., Chi Y., He B., Hao Y., Niu X., Huang B. (2024). Effects of intracellular Ca^2+^ on developmental potential and ultrastructure of cryopreserved-warmed oocyte in mouse. Cryobiology.

[49] Weiser A., Hermant A., Bermont F., Sizzano F., Karaz S., Alvarez-Illera P., Santo-Domingo J., Sorrentino V., Feige J.N., De Marchi U. (2023). The mitochondrial calcium uniporter (MCU) activates mitochondrial respiration and enhances mobility by regulating mitochondrial redox state. Redox Biol..

[50] Morimoto Y., Gamage U.S.K., Yamochi T., Saeki N., Morimoto N., Yamanaka M., Koike A., Miyamoto Y., Tanaka K., Fukuda A. (2023). Mitochondrial Transfer into Human Oocytes Improved Embryo Quality and Clinical Outcomes in Recurrent Pregnancy Failure Cases. Int. J. Mol. Sci..

[51] Winstanley Y.E., Liu J., Adhikari D., Gonzalez M.B., Russell D.L., Carroll J., Robker R.L. (2024). Dynamics of Mitochondrial DNA Copy Number and Membrane Potential in Mouse Pre-Implantation Embryos: Responses to Diverse Types of Oxidative Stress. Genes.

[52] Zhang H., Yan K., Sui L., Li P., Du Y., Hu J., Li M., Yang X., Liang X. (2021). Low-level pyruvate inhibits early embryonic development and maternal mRNA clearance in mice. Theriogenology.

[53] Li P., Zhang H., Yan K., Sui L., Du Y., Hu J., Xu H., Yang X., Liang X. (2022). Insufficient pyruvate in culture medium arrests mouse embryos at the first cleavage stage associated with abnormal epigenetic modifications. Theriogenology.

[54] Zhang T., Zheng Y., Han R., Kuang T., Min C., Wang H., Zhao Y., Wang J., Yang L., Che D. (2022). Effects of pyruvate on early embryonic development and zygotic genome activation in pigs. Theriogenology.

[55] Hong J., Tong H., Wang X., Lv X., He L., Yang X., Wang Y., Xu K., Liang Q., Feng Q. (2023). Embryonic diapause due to high glucose is related to changes in glycolysis and oxidative phosphorylation, as well as abnormalities in the TCA cycle and amino acid metabolism. Front. Endocrinol..

[56] Xiong Y.-Y., Zhu H.-Y., Shi R.-J., Wu Y.-F., Fan Y., Jin L. (2024). Regulation of glucose metabolism: Effects on oocyte, preimplantation embryo, assisted reproductive technology and embryonic stem cell. Heliyon.

[57] Da Fonseca Junior A.M., Ispada J., Dos Santos E.C., De Lima C.B., Da Silva J.V.A., Paulson E., Goszczynski D.E., Goissis M.D., Ross P.J., Milazzotto M.P. (2023). Adaptative response to changes in pyruvate metabolism on the epigenetic landscapes and transcriptomics of bovine embryos. Sci. Rep..

[58] Conaghan J., Handyside A.H., Winston R.M.L., Leese H.J. (1993). Effects of pyruvate and glucose on the development of human preimplantation embryos in vitro. Reproduction.

[59] Pawlak P., Lipinska P., Sell-Kubiak E., Kajdasz A., Derebecka N., Warzych E. (2024). Energy metabolism disorders during in vitro maturation of bovine cumulus-oocyte complexes interfere with blastocyst quality and metabolism. Dev. Biol..

[60] Bao S., Yin T., Liu S. (2024). Ovarian aging: Energy metabolism of oocytes. J. Ovarian Res..

[61] Imanaka S., Shigetomi H., Kobayashi H. (2022). Reprogramming of glucose metabolism of cumulus cells and oocytes and its therapeutic significance. Reprod. Sci..

[62] Okonkwo E., Saha B., Sahu G., Bera A., Sharma P. (2025). Blood-Based Lateral-Flow Immunoassays Dipstick Test for Damaged Mitochondrial Electron Transport Chain in Pyruvate Treated Rats with Combined Blast Exposure and Hemorrhagic Shock. J. Clin. Med..

[63] German H.M., Ciapaite J., Verhoeven-Duif N.M., Jans J.J.M. (2025). Anaplerosis by medium-chain fatty acids through complex interplay with glucose and glutamine metabolism. J. Biol. Chem..

[64] AL-Zubaidi U., Liu J., Cinar O., Robker R.L., Adhikari D., Carroll J. (2019). The spatio-temporal dynamics of mitochondrial membrane potential during oocyte maturation. Mol. Human Reprod..

[65] Trejo-Solís C., Serrano-García N., Castillo-Rodríguez R.A., Robledo-Cadena D.X., Jimenez-Farfan D., Marín-Hernández Á., Silva-Adaya D., Rodríguez-Pérez C.E., Gallardo-Pérez J.C. (2024). Metabolic dysregulation of tricarboxylic acid cycle and oxidative phosphorylation in glioblastoma. Rev. Neurosci..

[66] Casuso R.A. (2024). Mitochondrial puzzle in muscle: Linking the electron transport system to overweight. Obes. Rev..

[67] Chenna S., Koopman W.J.H., Prehn J.H.M., Connolly N.M.C. (2022). Mechanisms and mathematical modeling of ROS production by the mitochondrial electron transport chain. Am. J. Physiol.-Cell Physiol..

[68] Bahety D., Böke E., Rodríguez-Nuevo A. (2024). Mitochondrial morphology, distribution and activity during oocyte development. Trends Endocrinol. Metab..

[69] Fujii J., Homma T., Osaki T. (2022). Superoxide Radicals in the Execution of Cell Death. Antioxidants.

[70] Van Der Reest J., Nardini Cecchino G., Haigis M.C., Kordowitzki P. (2021). Mitochondria: Their relevance during oocyte ageing. Ageing Res. Rev..

[71] Kuznetsov A.V., Margreiter R., Ausserlechner M.J., Hagenbuchner J. (2022). The Complex Interplay between Mitochondria, ROS and Entire Cellular Metabolism. Antioxidants.

[72] Günther R., Pal A., Williams C., Zimyanin V.L., Liehr M., Von Neubeck C., Krause M., Parab M.G., Petri S., Kalmbach N. (2022). Alteration of Mitochondrial Integrity as Upstream Event in the Pathophysiology of SOD1-ALS. Cells.

[73] Wong H.-S., Mezera V., Dighe P., Melov S., Gerencser A.A., Sweis R.F., Pliushchev M., Wang Z., Esbenshade T., McKibben B. (2021). Superoxide produced by mitochondrial site IQ inactivates cardiac succinate dehydrogenase and induces hepatic steatosis in Sod2 knockout mice. Free Radic. Biol. Med..

[74] Boone C., Lewis S.C. (2024). Bridging lipid metabolism and mitochondrial genome maintenance. J. Biol. Chem..

[75] Giedt M.S., Thomalla J.M., White R.P., Johnson M.R., Lai Z.W., Tootle T.L., Welte M.A. (2023). Adipose triglyceride lipase promotes prostaglandin-dependent actin remodeling by regulating substrate release from lipid droplets. Development.

[76] (Han) Van Der Kolk J.H., Gross J.J., Gerber V., Bruckmaier R.M. (2017). Disturbed bovine mitochondrial lipid metabolism: A review. Vet. Q..

[77] Ruidas B. (2024). Mitochondrial lipid metabolism in metastatic breast cancer. Mitochondrial Commun..

[78] Kannan M., Lahiri S., Liu L.-K., Choudhary V., Prinz W.A. (2017). Phosphatidylserine synthesis at membrane contact sites promotes its transport out of the ER. J. Lipid Res..

[79] Panov A.V., Mayorov V.I., Dikalova A.E., Dikalov S.I. (2022). Long-Chain and Medium-Chain Fatty Acids in Energy Metabolism of Murine Kidney Mitochondria. Int. J. Mol. Sci..

[80] Wang Y., Palmfeldt J., Gregersen N., Makhov A.M., Conway J.F., Wang M., McCalley S.P., Basu S., Alharbi H., St. Croix C. (2019). Mitochondrial fatty acid oxidation and the electron transport chain comprise a multifunctional mitochondrial protein complex. J. Biol. Chem..

[81] Lee A.K., Tse A. (2005). Dominant Role of Mitochondria in Calcium Homeostasis of Single Rat Pituitary Corticotropes. Endocrinology.

[82] Pérez-Fuentes N., Alvariño R., Alfonso A., González-Jartín J., Vieytes M.R., Botana L.M. (2024). The Mode of Action of Enniatins A and B is Mediated by Interaction with SOC Reservoirs (A) and Mitochondrial Permeability Transition Pore (B). Expo. Health.

[83] Matuz-Mares D., González-Andrade M., Araiza-Villanueva M.G., Vilchis-Landeros M.M., Vázquez-Meza H. (2022). Mitochondrial Calcium: Effects of Its Imbalance in Disease. Antioxidants.

[84] Zhao G., Jia M., Zhu S., Ren H., Wang G., Xin G., Sun M., Wang X., Lin Q., Jiang Q. (2024). Mitotic ER-mitochondria contact enhances mitochondrial Ca^2+^ influx to promote cell division. Cell Rep..

[85] Decuypere J.-P., Welkenhuyzen K., Luyten T., Ponsaerts R., Dewaele M., Molgó J., Agostinis P., Missiaen L., De Smedt H., Parys J.B. (2011). Ins(1,4,5) P_3_ receptor-mediated Ca^2+^ signaling and autophagy induction are interrelated. Autophagy.

[86] Duxfield A., Munkley J., Briggs M.D., Dennis E.P. (2022). CRELD2 is a novel modulator of calcium release and calcineurin-NFAT signalling during osteoclast differentiation. Sci. Rep..

[87] Ziegler D.V., Vindrieux D., Goehrig D., Jaber S., Collin G., Griveau A., Wiel C., Bendridi N., Djebali S., Farfariello V. (2021). Calcium channel ITPR2 and mitochondria–ER contacts promote cellular senescence and aging. Nat. Commun..

[88] Zhou Y., Jing S., Liu S., Shen X., Cai L., Zhu C., Zhao Y., Pang M. (2022). Double-activation of mitochondrial permeability transition pore opening via calcium overload and reactive oxygen species for cancer therapy. J. Nanobiotechnol.

[89] Zhang I.X., Herrmann A., Leon J., Jeyarajan S., Arunagiri A., Arvan P., Gilon P., Satin L.S. (2023). ER stress increases expression of intracellular calcium channel RyR1 to modify Ca^2+^ homeostasis in pancreatic beta cells. J. Biol. Chem..

[90] Tang S., Wang X., Shen Q., Yang X., Yu C., Cai C., Cai G., Meng X., Zou F. (2015). Mitochondrial Ca^2+^ uniporter is critical for store-operated Ca^2+^ entry-dependent breast cancer cell migration. Biochem. Biophys. Res. Commun..

[91] De Ridder I., Kerkhofs M., Lemos F.O., Loncke J., Bultynck G., Parys J.B. (2023). The ER-mitochondria interface, where Ca2+ and cell death meet. Cell Calcium.

[92] Li X., Zhao X., Qin Z., Li J., Sun B., Liu L. (2025). Regulation of calcium homeostasis in endoplasmic reticulum–mitochondria crosstalk: Implications for skeletal muscle atrophy. Cell Commun. Signal.

[93] Sun C., Wang Q., Li P., Dong R., Lei Y., Hu Y., Yan Y., Song G. (2024). The ROS Mediates MCUb in Mitochondria-Regulated Apoptosis of TM4 Cells Induced by Titanium Dioxide Nanoparticles. Biol. Trace Elem. Res..

[94] Katona M., Bartók Á., Nichtova Z., Csordás G., Berezhnaya E., Weaver D., Ghosh A., Várnai P., Yule D.I., Hajnóczky G. (2022). Capture at the ER-mitochondrial contacts licenses IP3 receptors to stimulate local Ca^2+^ transfer and oxidative metabolism. Nat. Commun..

[95] Calvo-Rodriguez M., Hou S.S., Snyder A.C., Kharitonova E.K., Russ A.N., Das S., Fan Z., Muzikansky A., Garcia-Alloza M., Serrano-Pozo A. (2020). Increased mitochondrial calcium levels associated with neuronal death in a mouse model of Alzheimer’s disease. Nat. Commun..

[96] Parmar J., Von Jonquieres G., Gorlamandala N., Chung B., Craig A.J., Pinyon J.L., Birnbaumer L., Klugmann M., Moorhouse A.J., Power J.M. (2024). TRPC Channels Activated by G Protein-Coupled Receptors Drive Ca^2+^ Dysregulation Leading to Secondary Brain Injury in the Mouse Model. Transl. Stroke Res..

[97] Xue G., Li D., Wang Z., Liu Y., Yang J., Li C., Li X., Ma J., Zhang M., Lu Y. (2021). Interleukin-17 upregulation participates in the pathogenesis of heart failure in mice via NF-κB-dependent suppression of SERCA2a and Cav1.2 expression. Acta Pharmacol. Sin..

[98] Santulli G., Xie W., Reiken S.R., Marks A.R. (2015). Mitochondrial calcium overload is a key determinant in heart failure. Proc. Natl. Acad. Sci. USA.

[99] Almeida A., Delgado-Esteban M., Bolaños J.P., Medina J.M. (2002). Oxygen and glucose deprivation induces mitochondrial dysfunction and oxidative stress in neurones but not in astrocytes in primary culture. J. Neurochem..

[100] San-Millán I. (2023). The Key Role of Mitochondrial Function in Health and Disease. Antioxidants.

[101] Ng Y.S., Turnbull D.M. (2016). Mitochondrial disease: Genetics and management. J. Neurol..

[102] Carvalho F., Spier A., Chaze T., Matondo M., Cossart P., Stavru F. (2020). Listeria monocytogenes Exploits Mitochondrial Contact Site and Cristae Organizing System Complex Subunit Mic10 to Promote Mitochondrial Fragmentation and Cellular Infection. mBio.

[103] Lee Y., Lee S.-M., Choi J., Kang S., So S., Kim D., Ahn J.-Y., Jung H.-Y., Jeong J.-Y., Kang E. (2021). Mitochondrial DNA Haplogroup Related to the Prevalence of Helicobacter pylori. Cells.

[104] Nevoit G., Jarusevicius G., Potyazhenko M., Mintser O., Bumblyte I.A., Vainoras A. (2024). Mitochondrial Dysfunction and Risk Factors for Noncommunicable Diseases: From Basic Concepts to Future Prospective. Diseases.

[105] Cuanalo-Contreras K., Schulz J., Mukherjee A., Park K.-W., Armijo E., Soto C. (2023). Extensive accumulation of misfolded protein aggregates during natural aging and senescence. Front. Aging Neurosci..

[106] Li S.-Y., Gong X.-Y., Ndikuryayo F., Yang W.-C. (2025). The emerging role of oxygen redox in pathological progression of disorders. Ageing Res. Rev..

[107] Zhou Q., Ren C., Li J., Wang L., Duan Y., Yao R., Tian Y., Yao Y. (2024). The crosstalk between mitochondrial quality control and metal-dependent cell death. Cell Death Dis..

[108] Dominiak K., Galganski L., Budzinska A., Jarmuszkiewicz W. (2024). Coenzyme Q deficiency in endothelial mitochondria caused by hypoxia; remodeling of the respiratory chain and sensitivity to anoxia/reoxygenation. Free Radic. Biol. Med..

[109] Ma H., Guo X., Cui S., Wu Y., Zhang Y., Shen X., Xie C., Li J. (2022). Dephosphorylation of AMP-activated protein kinase exacerbates ischemia/reperfusion-induced acute kidney injury via mitochondrial dysfunction. Kidney Int..

[110] Hu L.-L., Liao M.-H., Liu Y.-X., Xing C.-H., Nong L.-L., Yang F.-L., Sun S.-C. (2024). Loss of AMPK activity induces organelle dysfunction and oxidative stress during oocyte aging. Biol. Direct.

[111] Sadria M., Layton A.T. (2021). Interactions among mTORC, AMPK and SIRT: A computational model for cell energy balance and metabolism. Cell Commun. Signal.

[112] Zong Y., Li H., Liao P., Chen L., Pan Y., Zheng Y., Zhang C., Liu D., Zheng M., Gao J. (2024). Mitochondrial dysfunction: Mechanisms and advances in therapy. Signal Transduct. Target. Ther..

[113] Kobayashi H., Imanaka S. (2025). Exploring potential pathways from oxidative stress to ovarian aging. J. Obstet. Gynaecol..

[114] Kasapoğlu I., Seli E. (2020). Mitochondrial Dysfunction and Ovarian Aging. Endocrinology.

[115] Han Y., Du Z., Wu H., Zhao R., Liu J., Gao S., Zeng S. (2025). CALB1 and RPL23 Are Essential for Maintaining Oocyte Quality and Function During Aging. Aging Cell.

[116] Chen Y., Zhang J., Tian Y., Xu X., Wang B., Huang Z., Lou S., Kang J., Zhang N., Weng J. (2024). Iron accumulation in ovarian microenvironment damages the local redox balance and oocyte quality in aging mice. Redox Biol..

[117] Vo K.C.T., Sato Y., Kawamura K. (2023). Improvement of oocyte quality through the SIRT signaling pathway. Reprod. Med. Biol..

[118] Kobayashi H., Imanaka S. (2024). Mitochondrial DNA Damage and Its Repair Mechanisms in Aging Oocytes. Int. J. Mol. Sci..

[119] An Z., Xie C., Lu H., Wang S., Zhang X., Yu W., Guo X., Liu Z., Shang D., Wang X. (2024). Mitochondrial Morphology and Function Abnormality in Ovarian Granulosa Cells of Patients with Diminished Ovarian Reserve. Reprod. Sci..

[120] Bertoldo M.J., Listijono D.R., Ho W.-H.J., Riepsamen A.H., Goss D.M., Richani D., Jin X.L., Mahbub S., Campbell J.M., Habibalahi A. (2020). NAD+ Repletion Rescues Female Fertility during Reproductive Aging. Cell Rep..

[121] Borsky P., Holmannova D., Andrys C., Kremlacek J., Fiala Z., Parova H., Rehacek V., Svadlakova T., Byma S., Kucera O. (2023). Evaluation of potential aging biomarkers in healthy individuals: Telomerase, AGEs, GDF11/15, sirtuin 1, NAD+, NLRP3, DNA/RNA damage, and klotho. Biogerontology.

[122] Xing X., Zhang J., Wu T., Zhang J., Wang Y., Su J., Zhang Y. (2021). SIRT1 reduces epigenetic and non-epigenetic changes to maintain the quality of postovulatory aged oocytes in mice. Exp. Cell Res..

[123] Zhang T., Zhou Y., Li L., Wang H.-H., Ma X.-S., Qian W.-P., Shen W., Schatten H., Sun Q.-Y. (2016). SIRT1, 2, 3 protect mouse oocytes from postovulatory aging. Aging.

[124] Pasquariello R., Ermisch A.F., Silva E., McCormick S., Logsdon D., Barfield J.P., Schoolcraft W.B., Krisher R.L. (2019). Alterations in oocyte mitochondrial number and function are related to spindle defects and occur with maternal aging in mice and humans†. Biol. Reprod..

[125] Adrian A.E., Liu T.T., Pascal L.E., Bauer S.R., DeFranco D.B., Ricke W.A. (2024). Aging-Related Mitochondrial Dysfunction Is Associated with Fibrosis in Benign Prostatic Hyperplasia. J. Gerontol. Ser. A.

[126] Lu J., Li H., Yu D., Zhao P., Liu Y. (2023). Heat stress inhibits the proliferation and differentiation of myoblasts and is associated with damage to mitochondria. Front. Cell Dev. Biol..

[127] Chen L., Thorup V.M., Kudahl A.B., Østergaard S. (2024). Effects of heat stress on feed intake, milk yield, milk composition, and feed efficiency in dairy cows: A meta-analysis. J. Dairy Sci..

[128] Jing J., Wang J., Xiang X., Yin S., Tang J., Wang L., Jia G., Liu G., Chen X., Tian G. (2024). Selenomethionine alleviates chronic heat stress-induced breast muscle injury and poor meat quality in broilers via relieving mitochondrial dysfunction and endoplasmic reticulum stress. Anim. Nutr..

[129] Jo J.-H., Nejad J.G., Kim H.-R., Lee H.-G. (2024). Effect of seven days heat stress on feed and water intake, milk characteristics, blood parameters, physiological indicators, and gene expression in Holstein dairy cows. J. Therm. Biol..

[130] Wrzecińska M., Kowalczyk A., Kordan W., Cwynar P., Czerniawska-Piątkowska E. (2023). Disorder of Biological Quality and Autophagy Process in Bovine Oocytes Exposed to Heat Stress and the Effectiveness of In Vitro Fertilization. Int. J. Mol. Sci..

[131] Guo Z., Gao S., Ouyang J., Ma L., Bu D. (2021). Impacts of Heat Stress-Induced Oxidative Stress on the Milk Protein Biosynthesis of Dairy Cows. Animals.

[132] Shi J., Ji Z., Yao X., Yao Y., Li C., Liang Q., Zhang X. (2024). HSP90 Enhances Mitophagy to Improve the Resistance of Car-Diomyocytes to Heat Stress in Wenchang Chickens. Int. J. Mol. Sci..

[133] Havalová H., Ondrovičová G., Keresztesová B., Bauer J.A., Pevala V., Kutejová E., Kunová N. (2021). Mitochondrial HSP70 Chaperone System—The Influence of Post-Translational Modifications and Involvement in Human Diseases. Int. J. Mol. Sci..

[134] Stamperna K., Giannoulis T., Dovolou E., Kalemkeridou M., Nanas I., Dadouli K., Moutou K., Mamuris Z., Amiridis G.S. (2021). Heat Shock Protein 70 Improves In Vitro Embryo Yield and Quality from Heat Stressed Bovine Oocytes. Animals.

[135] Gouda A., Tolba S., Mahrose K., Felemban S.G., Khafaga A.F., Khalifa N.E., Jaremko M., Moustafa M., Alshaharni M.O., Algopish U. (2024). Heat shock proteins as a key defense mechanism in poultry production under heat stress conditions. Poult. Sci..

[136] Suzuki N. (2023). Fine Tuning of ROS, Redox and Energy Regulatory Systems Associated with the Functions of Chloroplasts and Mitochondria in Plants under Heat Stress. Int. J. Mol. Sci..

[137] Lang L.I., Wang Z., Liu B., Chang-qing S.H.E.N., Jing-yi T.U., Shi-cheng W.A.N.G., Rui-ling L.E.I., Si-qi P.E.N.G., Xiong X.I.A.O., Yong-ju Z.H.A.O. (2024). The effects and mechanisms of heat stress on mammalian oocyte and embryo development. J. Therm. Biol..

[138] Zhang F.-L., Li W.-D., Zhu K.-X., Zhou X., Li L., Lee T.-L., Shen W. (2023). Aging-related aneuploidy is associated with mitochondrial imbalance and failure of spindle assembly. Cell Death Discov..

[139] Diao R.Y., Gustafsson Å.B. (2022). Mitochondrial quality surveillance: Mitophagy in cardiovascular health and disease. Am. J. Physiol.-Cell Physiol..

[140] Khan I., Mesalam A., Heo Y.S., Lee S.-H., Nabi G., Kong I.-K. (2023). Heat Stress as a Barrier to Successful Reproduction and Potential Alleviation Strategies in Cattle. Animals.

[141] Reddam A., McLarnan S., Kupsco A. (2022). Environmental Chemical Exposures and Mitochondrial Dysfunction: A Review of Recent Literature. Curr. Environ. Health Rep..

[142] Duarte-Hospital C., Tête A., Brial F., Benoit L., Koual M., Tomkiewicz C., Kim M.J., Blanc E.B., Coumoul X., Bortoli S. (2021). Mitochondrial Dysfunction as a Hallmark of Environmental Injury. Cells.

[143] Lim E.Y., Kim G.-D. (2024). Particulate Matter-Induced Emerging Health Effects Associated with Oxidative Stress and Inflammation. Antioxidants.

[144] Belyaeva E.A. (2023). Modulators of mitochondrial ATP-sensitive potassium channel affect cytotoxicity of heavy metals: Action on isolated rat liver mitochondria and AS-30D ascites hepatoma cells. Ecotoxicol. Environ. Saf..

[145] Choi S.-H., Ochirpurev B., Jo H.Y., Won J.-U., Toriba A., Kim H. (2023). Effects of polycyclic aromatic hydrocarbon exposure on mitochondrial DNA copy number. Hum. Exp. Toxicol..

[146] Jia Y., Li W., Li Y., Zhao L., Li C., Wang L., Fang J., Song S., Ji Y., Fang T. (2023). The Levels of Polycyclic Aromatic Hydrocarbons and Their Derivatives in Plasma and Their Effect on Mitochondrial DNA Methylation in the Oilfield Workers. Toxics.

[147] Guo L., Liu Z., Li P., Ji Y., Song S., Zheng N., Zhao L., Jia Y., Fang J., Wang H. (2022). Association between mitochondrial DNA methylation and internal exposure to polycyclic aromatic hydrocarbons (PAHs), nitrated-PAHs (NPAHs) and oxygenated-PAHs (OPAHs) in young adults from Tianjin, China. Ecotoxicol. Environ. Saf..

[148] Wang Y., Li S., Yang S., Li X., Liu L., Ma X., Niu D., Duan X. (2023). Exposure to phenanthrene affects oocyte meiosis by inducing mitochondrial dysfunction and endoplasmic reticulum stress. Cell Prolif..

[149] Lee Y., Cho J., Sohn J., Kim C. (2023). Health Effects of Microplastic Exposures: Current Issues and Perspectives in South Korea. Yonsei Med. J..

[150] Wu H., Feng L., Wu H., Wang L., Xu H., Fu F. (2025). Synergistic effects of PS-NPs and Cd on ovarian toxicity in adolescent rats: Ferroptosis by induction of mitochondrial redox imbalance via the SIRT3-SOD2/Gpx4 pathway. Ecotoxicol. Environ. Saf..

[151] Ahmad W., Alharthy R.D., Zubair M., Ahmed M., Hameed A., Rafique S. (2021). Toxic and heavy metals contamination assessment in soil and water to evaluate human health risk. Sci. Rep..

[152] Xiao C., Lai D. (2025). Impact of oxidative stress induced by heavy metals on ovarian function. J. Appl. Toxicol..

[153] Wrzecińska M., Kowalczyk A., Cwynar P., Czerniawska-Piątkowska E. (2021). Disorders of the Reproductive Health of Cattle as a Response to Exposure to Toxic Metals. Biology.

[154] Koyama H., Kamogashira T., Yamasoba T. (2024). Heavy Metal Exposure: Molecular Pathways, Clinical Implications, and Protective Strategies. Antioxidants.

[155] Shao C.-S., Zhou X.-H., Miao Y.-H., Wang P., Zhang Q.-Q., Huang Q. (2021). In situ observation of mitochondrial biogenesis as the early event of apoptosis. iScience.

[156] Kang X., Yan L., Wang J. (2024). Spatiotemporal Distribution and Function of Mitochondria in Oocytes. Reprod. Sci..

[157] Nicolson G.L. (2007). Metabolic syndrome and mitochondrial function: Molecular replacement and antioxidant supplements to prevent membrane peroxidation and restore mitochondrial function. J. Cell. Biochem..

[158] Paull D., Emmanuele V., Weiss K.A., Treff N., Stewart L., Hua H., Zimmer M., Kahler D.J., Goland R.S., Noggle S.A. (2013). Nuclear genome transfer in human oocytes eliminates mitochondrial DNA variants. Nature.

[159] Sloan D.B., Fields P.D., Havird J.C. (2015). Mitonuclear linkage disequilibrium in human populations. Proc. R. Soc. B..

[160] Tachibana M., Amato P., Sparman M., Woodward J., Sanchis D.M., Ma H., Gutierrez N.M., Tippner-Hedges R., Kang E., Lee H.-S. (2013). Towards germline gene therapy of inherited mitochondrial diseases. Nature.

[161] Gómez-Tatay L., Hernández-Andreu J., Aznar J. (2017). Mitochondrial Modification Techniques and Ethical Issues. J. Clin. Med..

[162] Pasquariello R., Verdile N., Brevini T.A.L., Gandolfi F., Boiti C., Zerani M., Maranesi M. (2020). The Role of Resveratrol in Mammalian Reproduction. Molecules.

[163] Jiang T., Chen Y., Gu X., Miao M., Hu D., Zhou H., Chen J., Teichmann A.T., Yang Y. (2023). Review of the Potential Therapeutic Effects and Molecular Mechanisms of Resveratrol on Endometriosis. Int. J. Women’s Health.

[164] Yen G.-C., Duh P.-D., Lin C.-W. (2003). Effects of Resveratrol and 4-hexylresorcinol on Hydrogen Peroxide-induced Oxidative DNA Damage in Human Lymphocytes. Free Radic. Res..

[165] Novakovic R., Rajkovic J., Gostimirovic M., Gojkovic-Bukarica L., Radunovic N. (2022). Resveratrol and Reproductive Health. Life.

[166] Zaychenko G., Stryga O., Sinitsyna O., Doroshenko A., Sulaieva O., Falalyeyeva T., Kobyliak N. (2022). Resveratrol Effects on the Reproductive System in Ovariectomized Rats: Deciphering Possible Mechanisms. Molecules.

[167] Bartra C., Yuan Y., Vuraić K., Valdés-Quiroz H., Garcia-Baucells P., Slevin M., Pastorello Y., Suñol C., Sanfeliu C. (2024). Resveratrol Activates Antioxidant Protective Mechanisms in Cellular Models of Alzheimer’s Disease Inflammation. Antioxidants.

[168] Taherian M., Norenberg M.D., Panickar K.S., Shamaladevi N., Ahmad A., Rahman P., Jayakumar A.R. (2020). Additive Effect of Resveratrol on Astrocyte Swelling Post-exposure to Ammonia, Ischemia and Trauma In Vitro. Neurochem. Res..

[169] Omraninava M., Razi B., Aslani S., Imani D., Jamialahmadi T., Sahebkar A. (2021). Effect of resveratrol on inflammatory cytokines: A meta-analysis of randomized controlled trials. Eur. J. Pharmacol..

[170] Chuang Y.-C., Chen S.-D., Hsu C.-Y., Chen S.-F., Chen N.-C., Jou S.-B. (2019). Resveratrol Promotes Mitochondrial Biogenesis and Protects against Seizure-Induced Neuronal Cell Damage in the Hippocampus Following Status Epilepticus by Activation of the PGC-1α Signaling Pathway. Int. J. Mol. Sci..

[171] Nishigaki A., Tsubokura H., Tsuzuki-Nakao T., Okada H. (2022). Hypoxia: Role of SIRT1 and the protective effect of resveratrol in ovarian function. Reprod. Med. Biol..

[172] Jiang X., Ma Y., Gong S., Zi X., Zhang D. (2024). Resveratrol Promotes Proliferation, Antioxidant Properties, and Progesterone Production in Yak (*Bos grunniens*) Granulosa Cells. Animals.

[173] Xu G., Dong Y., Wang Z., Ding H., Wang J., Zhao J., Liu H., Lv W. (2023). Melatonin Attenuates Oxidative Stress-Induced Apoptosis of Bovine Ovarian Granulosa Cells by Promoting Mitophagy via SIRT1/FoxO1 Signaling Pathway. Int. J. Mol. Sci..

[174] Sugiyama M., Kawahara-Miki R., Kawana H., Shirasuna K., Kuwayama T., Iwata H. (2015). Resveratrol-induced mitochondrial synthesis and autophagy in oocytes derived from early antral follicles of aged cows. J. Reprod. Dev..

[175] Wang J., Jia R., Celi P., Zhuo Y., Ding X., Zeng Q., Bai S., Xu S., Yin H., Lv L. (2022). Resveratrol Alleviating the Ovarian Function Under Oxidative Stress by Alternating Microbiota Related Tryptophan-Kynurenine Pathway. Front. Immunol..

[176] Okamoto N., Sato Y., Kawagoe Y., Shimizu T., Kawamura K. (2022). Short-term resveratrol treatment restored the quality of oocytes in aging mice. Aging.

[177] Higashida K., Kim S.H., Jung S.R., Asaka M., Holloszy J.O., Han D.-H. (2013). Effects of Resveratrol and SIRT1 on PGC-1α Activity and Mitochondrial Biogenesis: A Reevaluation. PLoS Biol..

[178] Nishigaki A., Kido T., Kida N., Kakita-Kobayashi M., Tsubokura H., Hisamatsu Y., Okada H. (2020). Resveratrol protects mitochondrial quantity by activating SIRT1/PGC-1α expression during ovarian hypoxia. Reprod. Med. Biol..

[179] Zou W., Wang X., Xia X., Zhang T., Nie M., Xiong J., Fang X. (2024). Resveratrol protected against the development of endometriosis by promoting ferroptosis through miR-21-3p/p53/SLC7A11 signaling pathway. Biochem. Biophys. Res. Commun..

[180] Sun X., Fu P., Xie L., Chai S., Xu Q., Zeng L., Wang X., Jiang N., Sang M. (2020). Resveratrol inhibits the progression of cervical cancer by suppressing the transcription and expression of HPV E6 and E7 genes. Int. J. Mol. Med..

[181] Della Corte L., Noventa M., Ciebiera M., Magliarditi M., Sleiman Z., Karaman E., Catena U., Salvaggio C., Falzone G., Garzon S. (2020). Phytotherapy in endometriosis: An up-to-date review. J. Complement. Integr. Med..

[182] Wang W., Zhou M., Xu Y., Peng W., Zhang S., Li R., Zhang H., Zhang H., Cheng S., Wang Y. (2021). Resveratrol-Loaded TPGS-Resveratrol-Solid Lipid Nanoparticles for Multidrug-Resistant Therapy of Breast Cancer: In Vivo and In Vitro Study. Front. Bioeng. Biotechnol..

[183] Bezerra M.É.S., Gouveia B.B., Barberino R.S., Menezes V.G., Macedo T.J.S., Cavalcante A.Y.P., Monte A.P.O., Santos J.M.S., Matos M.H.T. (2018). Resveratrol promotes in vitro activation of ovine primordial follicles by reducing DNA damage and enhancing granulosa cell proliferation via phosphatidylinositol 3-kinase pathway. Reprod. Domest. Anim..

[184] Li N., Liu L. (2018). Mechanism of resveratrol in improving ovarian function in a rat model of premature ovarian insufficiency. J. Obstet. Gynaecol..

[185] Ragonese F., Monarca L., De Luca A., Mancinelli L., Mariani M., Corbucci C., Gerli S., Iannitti R.G., Leonardi L., Fioretti B. (2021). Resveratrol depolarizes the membrane potential in human granulosa cells and promotes mitochondrial biogenesis. Fertil. Steril..

[186] Li R., Li E., Kamili G., Ou S., Yang D. (2022). Effect of resveratrol on superovulation in mice. Biomed. Pharmacother..

[187] Gutierrez-Castillo E., Diaz F.A., Talbot S.A., Bondioli K.R. (2023). Effect of bovine oocyte vitrification with EGTA and post-warming recovery with resveratrol on meiotic spindle, mitochondrial function, reactive oxygen species, and developmental competence. Theriogenology.

[188] Zheng L., Luo Y., Zhou D., Liu H., Zhou G., Meng L., Hou Y., Liu C., Li J., Fu X. (2023). Leonurine improves bovine oocyte maturation and subsequent embryonic development by reducing oxidative stress and improving mitochondrial function. Theriogenology.

[189] Zhang Y., Guo W., Wen Y., Xiong Q., Liu H., Wu J., Zou Y., Zhu Y. (2012). SCM-198 attenuates early atherosclerotic lesions in hypercholesterolemic rabbits via modulation of the inflammatory and oxidative stress pathways. Atherosclerosis.

[190] Ma W., Zhao X., Wang Q., Wu X., Yang T., Chen Y., Zhu Y., Wang X. (2024). SCM-198 ameliorates the quality of postovulatory and maternally aged oocytes by reducing oxidative stress. J. Ovarian Res..

[191] Shao Y., Luo Y., Sun Y., Jiang J., Li Z., Wang Z., Wang M., Gu X. (2024). Leonurine Exerts Anti-Inflammatory Effects in Lipopolysaccharide (LPS)-Induced Endometritis by Modulating Mouse JAK-STAT/PI3K-Akt/PPAR Signaling Pathways. Genes.

[192] Luo D., Zhang J.-B., Liu W., Yao X., Guo H., Jin Z.-L., Zhang M.-J., Yuan B., Jiang H., Kim N.-H. (2020). Leonurine improves in vitro porcine embryo development competence by reducing reactive oxygen species production and protecting mitochondrial function. Theriogenology.

[193] Chi Y.-N., Hai D.-M., Ma L., Cui Y.-H., Hu H.-T., Liu N., Juan-Du, Lan X.-B., Yu J.-Q., Yang J.-M. (2023). Protective effects of leonurine hydrochloride on pyroptosis in premature ovarian insufficiency via regulating NLRP3/GSDMD pathway. Int. Immunopharmacol..

[194] Yang D., Jia W., Zhu Y.Z. (2016). Leonurine, a Potential Agent of Traditional Chinese Medicine: Recent Updates and Future Perspectives. Nat. Product. Commun..

[195] Xu W., Cui J., Zhou F., Bai M., Deng R., Wang W. (2020). Leonurine protects against dexamethasone-induced cytotoxicity in pancreatic β-cells via PI3K/Akt signaling pathway. Biochem. Biophys. Res. Commun..

[196] Qu J., Hu H., Niu H., Sun X., Li Y. (2023). Melatonin restores the declining maturation quality and early embryonic development of oocytes in aged mice. Theriogenology.

[197] Zhang H., Li C., Wen D., Li R., Lu S., Xu R., Tang Y., Sun Y., Zhao X., Pan M. (2022). Melatonin improves the quality of maternally aged oocytes by maintaining intercellular communication and antioxidant metabolite supply. Redox Biol..

[198] Cho J.H., Bhutani S., Kim C.H., Irwin M.R. (2021). Anti-inflammatory effects of melatonin: A systematic review and meta-analysis of clinical trials. Brain Behav. Immun..

[199] Wu G.-Q., Jia B.-Y., Li J.-J., Fu X.-W., Zhou G.-B., Hou Y.-P., Zhu S.-E. (2011). L-carnitine enhances oocyte maturation and development of parthenogenetic embryos in pigs. Theriogenology.

[200] Li J.-M., Zhang Z., Kong A., Lai W., Xu W., Cao X., Zhao M., Li J., Shentu J., Guo X. (2023). Dietary L-carnitine regulates liver lipid metabolism *via* simultaneously activating fatty acid *β*-oxidation and suppressing endoplasmic reticulum stress in large yellow croaker fed with high-fat diets. Br. J. Nutr..

[201] Reader K.L., Cox N.R., Stanton J.-A.L., Juengel J.L. (2015). Effects of acetyl-L-carnitine on lamb oocyte blastocyst rate, ultrastructure, and mitochondrial DNA copy number. Theriogenology.

[202] Li J., Liu L., Weng J., Yin T., Yang J., Feng H.L. (2021). Biological roles of L-carnitine in oocyte and early embryo development. Mol. Reprod. Dev..

[203] Lee Y.-C.G., Chou H.-C., Chen Y.-T., Tung S.-Y., Ko T.-L., Buyandelger B., Wen L.-L., Juan S.-H. (2022). l-Carnitine reduces reactive oxygen species/endoplasmic reticulum stress and maintains mitochondrial function during autophagy-mediated cell apoptosis in perfluorooctanesulfonate-treated renal tubular cells. Sci. Rep..

[204] Virmani M.A., Cirulli M. (2022). The Role of l-Carnitine in Mitochondria, Prevention of Metabolic Inflexibility and Disease Initiation. Int. J. Mol. Sci..

[205] Modak A.K., Alam M.H., Islam M.N., Paul N., Akter I., Hashem M.A., Kabir A.A., Moniruzzaman M. (2022). L-Carnitine Supports the In Vitro Growth of Buffalo Oocytes. Animals.

[206] Catandi G.D., Cheng M.-H., Chicco A.J., Chen T., Carnevale E.M. (2023). L-carnitine enhances developmental potential of bovine oocytes matured under high lipid concentrations in vitro. Anim. Reprod. Sci..

[207] López-Sánchez C., De Andrés F., Zougagh M., Ríos Á. (2025). A multi-step approach for the accurate screening and determination of Coenzyme Q10 (Nano)micelles. Anal. Chim. Acta.

[208] Ma L., Li X., Li C., Chen P., Lan Y., Huang Y., Xu W., Zhou J. (2023). Association of Coenzyme Q10 with Premature Ovarian Insufficiency. Reprod. Sci..

[209] Cao S., Yan H., Tang W., Zhang H., Liu J. (2023). Effects of dietary coenzyme Q10 supplementation during gestation on the embryonic survival and reproductive performance of high-parity sows. J. Anim. Sci. Biotechnol..

[210] McRae M.P. (2023). Coenzyme Q10 Supplementation in Reducing Inflammation: An Umbrella Review. J. Chiropr. Med..

[211] Zhang L., Li Y., Hu J., Liu Z. (2022). Overexpression of enzymes in glycolysis and energy metabolic pathways to enhance coenzyme Q10 production in Rhodobacter sphaeroides VK-2-3. Front. Microbiol..

[212] Yen H.-C., Yeh W.-Y., Lee S.-H., Feng Y.-H., Yang S.-L. (2020). Characterization of human mitochondrial PDSS and COQ proteins and their roles in maintaining coenzyme Q10 levels and each other’s stability. Biochim. Biophys. Acta (BBA)-Bioenerg..

[213] Niu Y.-J., Zhou W., Nie Z.-W., Zhou D., Xu Y.-N., Ock S.A., Yan C.-G., Cui X.-S. (2020). Ubiquinol-10 delays postovulatory oocyte aging by improving mitochondrial renewal in pigs. Aging.

[214] Yang C.-X., Liu S., Miao J.-K., Mou Q., Liu X.-M., Wang P.-C., Huo L.-J., Du Z.-Q. (2021). CoQ10 improves meiotic maturation of pig oocytes through enhancing mitochondrial function and suppressing oxidative stress. Theriogenology.

[215] Heydarnejad A., Ostadhosseini S., Varnosfaderani S.R., Jafarpour F., Moghimi A., Nasr-Esfahani M.H. (2019). Supplementation of maturation medium with CoQ10 enhances developmental competence of ovine oocytes through improvement of mitochondrial function. Mol. Reprod. Dev..

[216] Tsui K.-H., Li C.-J. (2023). Mitoquinone shifts energy metabolism to reduce ROS-induced oxeiptosis in female granulosa cells and mouse oocytes. Aging.

